# Dataset to evaluate the geology, metamorphic conditions and pseudosection modeling of the Luswishi Dome, Copperbelt, Zambia

**DOI:** 10.1016/j.dib.2021.107525

**Published:** 2021-10-29

**Authors:** Kabang'u G. Sakuwaha, Toru Takeshita, Ahmed H. Ahmed

**Affiliations:** a Department of Natural History Sciences, Graduate School of Science, Hokkaido University, Sapporo 060-0808, Japan; b Department of Geology, School of Mines, University of Zambia, P.O Box 32379, Lusaka, Zambia

**Keywords:** Lufilian Arc, Luswishi Dome, Copperbelt, Zambia, Geothermobarometry, Pseudosection

## Abstract

This Data in Brief article presents methods used to acquire and process the data presented and discussed in a research paper co-submitted to the Journal of African Earth Sciences titled: “Comparative geology and metamorphic evolution of the Luswishi Dome, Copperbelt, Zambia: Implications for exploration targeting.” [1]. The dataset includes geographical coordinates, lithological logs of drill holes, rock and mineral chemistry data, and pseudosections. The dataset is hosted in the Mendeley Data repository in relevant sub-folders as supplementary data to the research paper. In order to provide spatial context for the study area map, geographical coordinates and collar information of the studied drill holes obtained from secondary sources are also provided in tabular form. Lithological logs summarize physical characteristics of lithological observations made on drill core. Rock chemistry data were obtained by fusion X-ray fluorescence**,** XRF (outsourced to the Actlabs, Canada), and mineral chemistry data were obtained by electron probe microanalyzer, EPMA, model JXA-8800R at Hokkaido University in Japan. Pseudosections were computed using Perple_X 6.8.6 software, as a function of rock compositions, temperature and pressure following the methods outlined in the online Perple_X documentation files available on the developer's website. The data maybe of potential use for improving our understanding of the geology, geochemical characteristics, and metamorphic conditions of the study area, with potential to inform further research and explorations works in the Dome's region of the Lufilian Arc.

## Specifications Table

**Table:** 

Subject	Earth and Planetary Sciences
Specific subject area	Geochemistry and metamorphic geology
Type of data	Table Image (TIF, JPG files) Figure Text files Database- (XLS)
How data were acquired	1. Literature search (coordinates and maps)2. Geological field work (drill core logging)3. Laboratory analyses (petrography, rock, and mineral chemistry)
Data format	Raw Analyzed Filtered Secondary Processed
Parameters for data collection	Lithologic logging considered available drill core from both historical and recent drilling campaigns conducted between 1960-2018. All drill core is available on request at the core repository in Kalulushi town, Copperbelt Province. The original data sources are hosted by the Ministry of Mines, Zambia. Our sampling focused on lithologic intervals with diagnostic minerals useful for geothermobarometry applications.
Description of data collection	Mineral chemical data were obtained using the JEOL JXA 8800R electron microprobe analyzer (EPMA) at Hokkaiso University. The equipment was operated at 15 keV acceleration voltage and 12 nA beam current. The data were regressed using the ZAF oxide matrix correction program, a combination of corrections for the atomic number effect (Z), absorption (A), and fluorescence phenomena (F), supplied by JEOL Limited.
Data source location	1. Primary data sources are hosted by the Ministry of Mines and Minerals Development, in Lusaka Zambia and the Zambia Consolidated Copper Mines-Investment Holdings (ZCCM-IH) archives in Kalulushi town, Copperbelt Province.2. Data collected for purposes of related research paper are stored online.
Data accessibility	Repository name: Mendeley Data Data identification number: DOI: 10.17632/bdtfd4ksrr.1 Direct URL to data: http://dx.doi.org/10.17632/bdtfd4ksrr.1
Related research article	Sakuwaha, K. G., Takeshita, T., Ahmed, A. H., 2021 Comparative geology and metamorphic evolution of the Luswishi Dome, Copperbelt, Zambia: Implications for exploration targeting. Journal of African Earth Science. Published. DOI: 10.1016/j.jafrearsci.2021.104349

## Value of the Data

• These data enable partial reconstruction of the stratigraphy of the study area and geothermobarometry calculations, which are useful for geologic correlations and comparisons to other parts of the region which host sulfide ore mineralization.
• These data are relevant for Copperbelt geologists, explorers, and researchers. The dataset may also be useful for educational purposes.
• The data might be used to inform future research and exploration works around the Luswishi Dome and potentially the entire Copperbelt region.

## 1 Data Description

The dataset described herein include lithologic logs, rock, and mineral chemistry data, and phase equilibria diagrams (pseudosections) calculated for four garnet-bearing pelitic schists and one kyanite-mica schist. Lithologic logs consist of interval data summarizing observations made on the drill core. Where possible the intervals represent actual depths from the surface, whereas estimates were made for portions where core intervals were missing due to physical damage of old core trays. The lithological logs are available in excel format (drill logs) in Mendeley Data here. Rock chemistry data obtained by fusion XRF are also provided as a filtered excel file titled “whole rock geochemical data in the same folder in Mendeley Data. The data shows oxide compositions (in weight. %) of six samples used in the related paper to evaluate the metamorphic evolution of the study area. In the second tab of the excel sheet, whole rock compositions are recalculated on a molar basis after adjusting the weight % amount of CaO equal to 3.33 times that of P_2_O_5_, subtracted from the XRF composition to account for the presence of apatite in samples containing apatite after (Weller et al., 2015 and references therein) [2]. This adjustment is done by assuming that apatite is the only phosphorous bearing mineral hence, P_2_O_3_ is removed from the bulk composition and CaO proportionally adjusted to account for the contribution of apatite [Ca_5_(PO4)_3_(OH, F, Cl)]. This is necessary because apatite cannot be reproduced by phase equilibria modelling due to lack of suitable endmember thermodynamic data in the utilized thermodynamic dataset.

Mineral chemistry data is given as an excel database which shows the chemical compositions of minerals obtained by electron microprobe (EPMA). EPMA analyses were performed at operating conditions of 15 keV accelerating voltage and 12 nA beam current with natural oxides as standards. Detailed analytical techniques are described in the related paper. The data is presented as groups of minerals, with cations per formular units (cpfu) calculated on the basis of appropriate oxygen numbers as described in Deer et al. (2013) [3] on separate tabs (named “Major minerals_all.xls). Additionally, rim-to-rim quantitative EPMA chemical compositions (along profile lines) of representative garnets in the studied samples are given to supplement the associated figures presented in the related research article (Figures 8, 9 and 10). Lastly, a folder labeled “Pseudosections”, contains sub-folders within it labeled by sample name (D440, D452, D472, D482 and D490). Each sub-folder contains files generated by Perple_X software (Connolly, 2005) [4], version 6.8.6. The files include a project definition file, a .dat file (my_project) which is the input file for pseudosection calculations. Calculation output files (.ps) are the outputs which can be opened and edited using any graphical softwares such as GIMP, Adobe illustrator, Corel Draw etc. Images and annotated figures (TIF or JPG) in these sub-folders represent processed .ps files (using Adobe illustrator).

## 2 Experimental Design, Materials and Methods

### 2.1 Geology of the study area

The Luswishi Dome is located in the Dome's region of Lufilian Arc, a tectonic belt which hosts copper orebodies of the Central African Copperbelt. The geology of the study area consists of a Precambrian basement core, rimmed by Neoproterpzoic metasediments and carbonates which were deformed and metamorphosed during the Pan-African Lufilian Orogeny. Lack of outcrop exposures hampers detailed geologic mapping, but exploration drilling around the area since the early 1960’s anable research. A number of drill holes (Table 1) were selected for lithologic logging and sampling. The study area map and cross sections are presented in the related research article (Figures 1, 3 and 4).

**Table 1:** GPS Coordinates of the studied drill holes.

Drillhole_ ID	Easting (UTM_WGS84)	Northing (UTM_WGS84)	Total Depth (m)	Inclination	Azimuth	Elevation (m)
CL 87	517588	8626142	569.06	−90	0	1336
CL 89	517597	8626000	394.71	−90	0	1340
CL 102	509882	8622185	334.97	−70	135	1336
LCP-MUJI-DH0002	507350	8622498	135.5	−70	90	1311
LCP-MUJI-DH0003	517545	8626002	312	−70	90	1342
LCP-MUJI-DH0005	509407	8621268	94.5	−70	160	1351
MP 490	485330	8591403	457.5	0	0	1286
CZSE0001	540970	8563952	403.55	−80	220	1215
CZSE0012	542787	8560767	169.84	−70	315	1214
CZSE0014	542059	8561337	302.5	−70	0	1218
CZCD0005	541817	8583097	865.4	−80	38	1237
CZSE0015	540033	8562796	245.5	−70	270	1191
CZCD0010	541611	8575275	204	−60	160	1196
CZCD0011	538239	8583230	251.8	−60	330	1175
ZNADD001	509612	8629307	400.3	−50	222	1310
ZNADD002	509736	8629124	299.3	−50	225	1307
ZNADD003	510552	8629310	245.3	−50	235	1303
ZNADD004	511122	8629233	292.8	−50	238	1347
ZNADD005	510614	8628857	353.3	−50	50	1346

### 2.2 Data collection

Drill core sampling focused on lithology intervals with diagnostic metamorphic minerals useful for geothermobarometry. Quarter core samples measuring approximately 10 cm each were cut and collected for laboratory analyses. Normal thin sections were prepared for petrographic examinations, after which samples with suitable mineral compositions were selected for chemical analyses (XRF and EPMA). XRF analyses including QAQC were (outsourced to the Activation laboratories in Canada) were performed on the same rock slabs from which thin sections were cut. Rock chemistry data were then recalculated on a molar basis for input in thermodynamic (pseudosection) modeling.

### 2.3 Data processing

Methods of pseudosection calculations draw heavily on the Perple_X tutorials and manuals hosted on the developer's website [5]. In summary, pseudosections for the studied samples were calculated to show possible mineral assemblages over a range of temperature and pressure, *P-T* conditions for the given bulk rock compositions in chemical systems involving mineral solid solutions. Derivations of system components were performed as outlined by Tinkham et al. (2001) [6]. Mineral pseudocompounds and *P-T* paths appropriate to each specific sample were identified by integrating theoretical thermobarometry calculations, inspection of the pseudosections and comparisons to the mineral assemblages and textures observed in thin section. Variations in modal amounts of mineral phases (volume. %) were accomplished by using the Perple_X subprogram *werami*, operational mode 2 (properties on a 2d grid), property 7 (Mode vol of a phase). Similarly, compositional isopleths for garnet and plagioclase (where applicable) were calculated using *werami*, operation mode 2, property 8 (mole composition of a phase). For garnet, compositions were defined in terms of solid solution endmembers (pyrope, almandine, spessartine, and grossular), whereas the ratio Fe/ (Mg +Mg) was defined in terms of system components (MgO and FeO). For plagioclase, compositional isopleths were defined in terms of system components (CaO, Na_2_O, and K_2_O). Calculation output files (.tab) for isomode and isopleths were plotted using Pywerami, a free software availablehere. Images generated were saved as .jpg files and .ps files which were then modified to make them easier to read and overlain on the appropriate pseudosections using adobe illustrator. The interested reader is referred to comprehensive tutorials on the perplex website https://www.perplex.ethz.ch.

## CRediT Author Statement

**Kabang'u G. Sakuwaha:** Conceptualization, field data collection, analytical works, interpretations and manuscript writing, **Takeshita Toru**: Supervision, reviewing and editing of manuscript. **Ahmed H. Ahmed**: Conceptualization and field work support.

## Declaration of Competing Interest

(1) All third-party financial support for the work this article; None
(2) All financial relationships with any entity that could be viewed as relevant to data described in this manuscript; None
(3) All sources of revenue with relevance to this work where payments have been made to authors, or their institutions on their behalf, within the 36 months prior to submission; Japan International Cooperation Agency, (JICA), through the Kizuna scholarship program (Human Resources Development for the mining sector).
(4) Any other interactions with the sponsor, outside of the submitted work; continued scholarship for PhD research work at Tsukuba University, Japan to Kabang'u G. Sakuwaha
(5) Any relevant patents or copyrights (planned, pending, or issued); none
(6) Any other relationships or affiliations that may be perceived by readers to have influenced, or give the appearance of potentially influencing, what has been written in this article. None
  The authors declare that they have no known competing financial interests or personal relationships which have or could be perceived to have influenced the work reported in this article. [1].

## Appendix Supplementary materials

Image, application 1

Image, application 2

Image, application 3

Image, application 4

Image, application 5
