# Supplementary figures and images for: Dataset to evaluate the geology, metamorphic conditions and pseudosection modeling of the Luswishi Dome, Copperbelt, Zambia
Source: Data Brief. 2021 Oct 29;39:107525. doi: 10.1016/j.dib.2021.107525 (PMC8586738; doi:10.1016/j.dib.2021.107525)

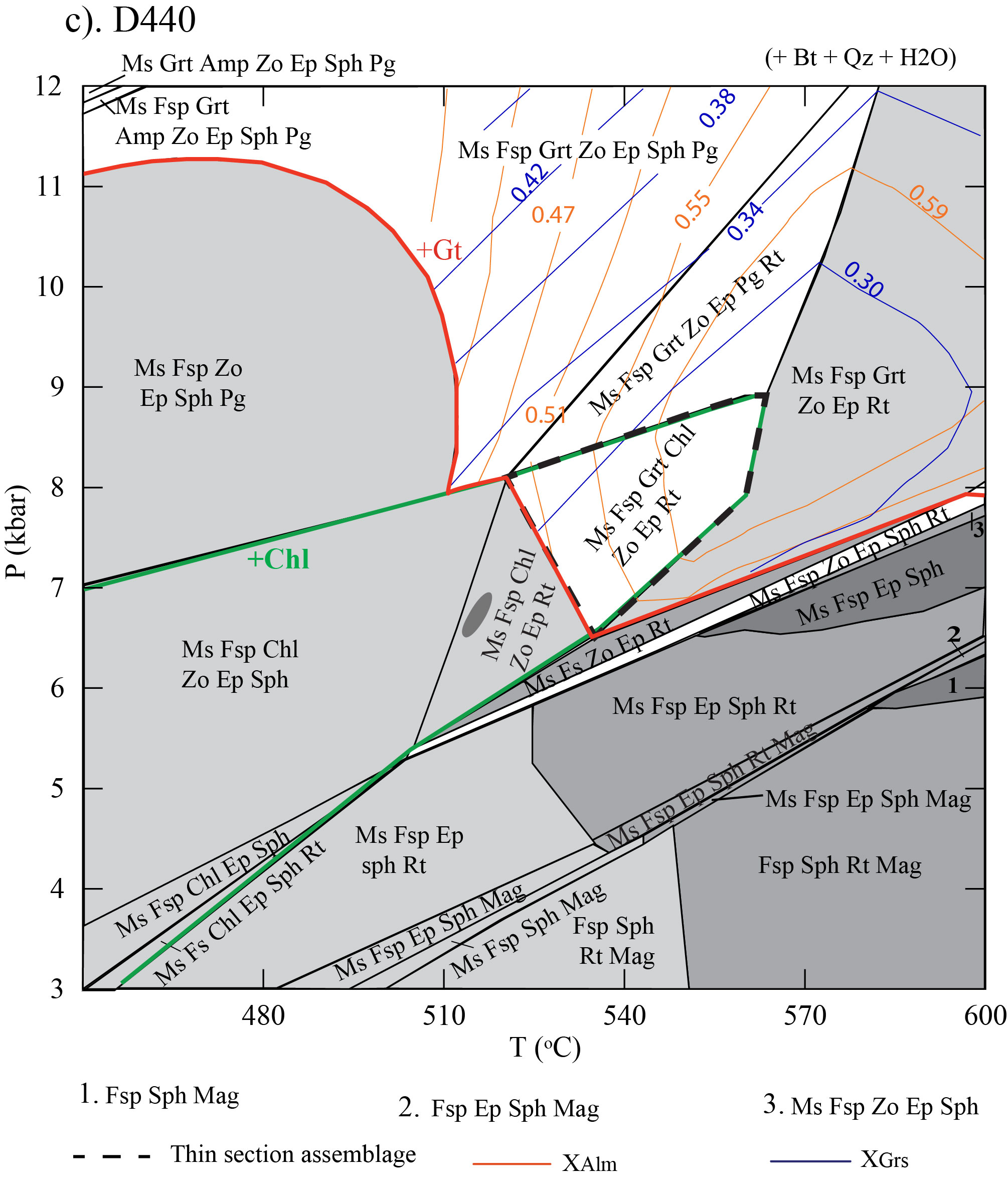

Supplement: Supplementary file 4 [file mmc4.zip › Pseudosections/Pseudosections/D440/D440.jpg]

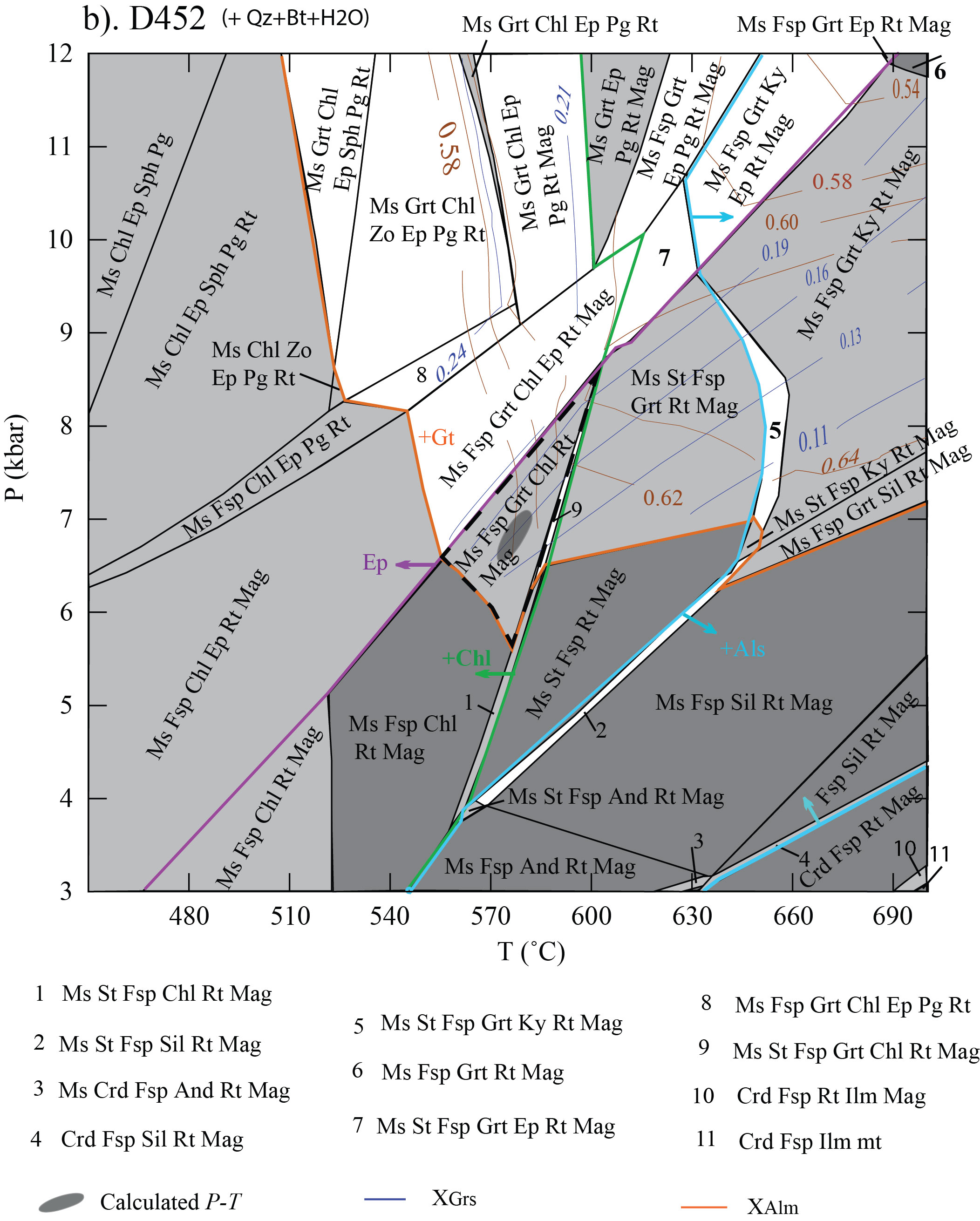

Supplement: Supplementary file 4 [file mmc4.zip › Pseudosections/Pseudosections/D452/D452.jpg]

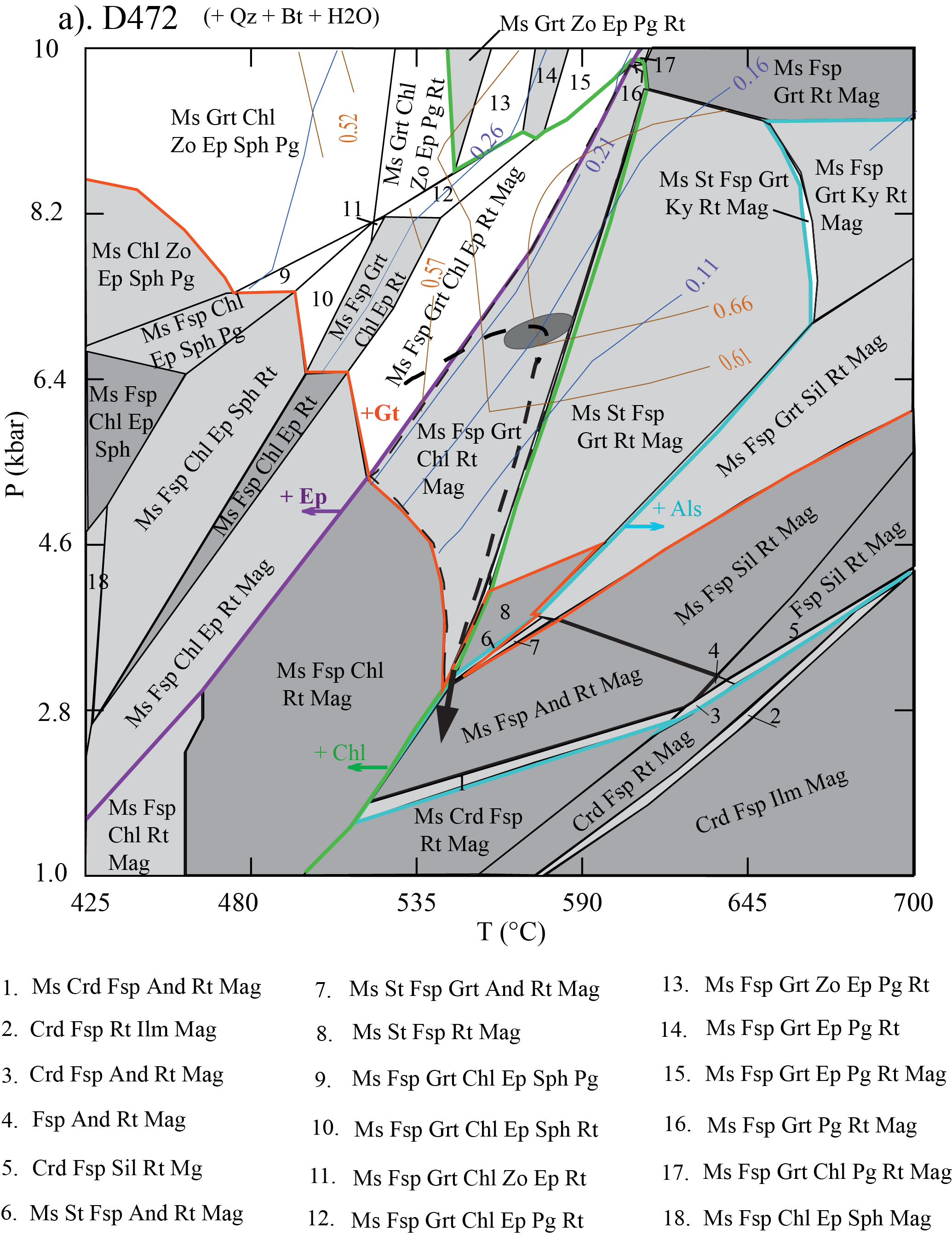

Supplement: Supplementary file 4 [file mmc4.zip › Pseudosections/Pseudosections/D472/D472.jpg]

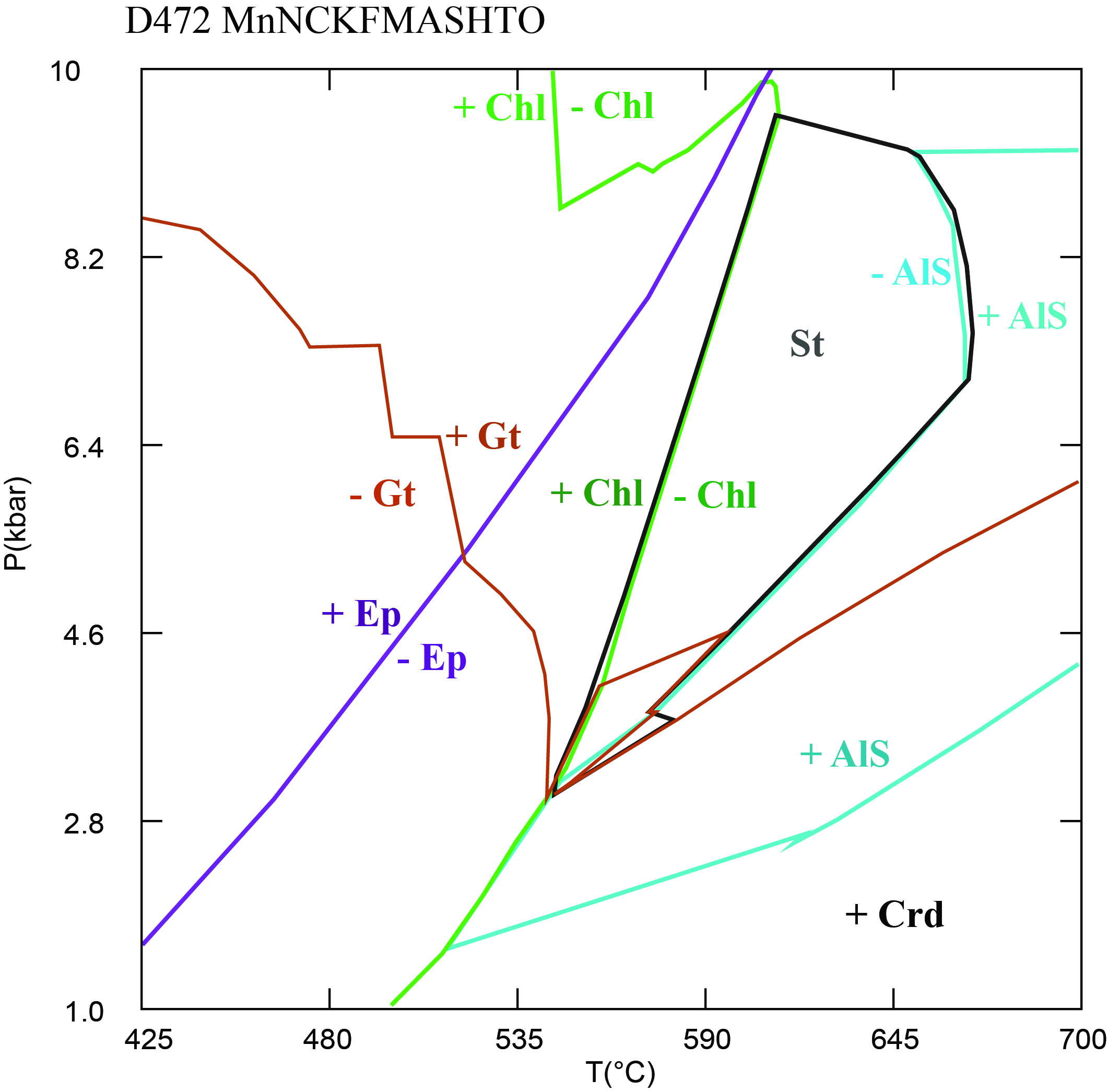

Supplement: Supplementary file 4 [file mmc4.zip › Pseudosections/Pseudosections/D472/D472-Main rxns.tif]

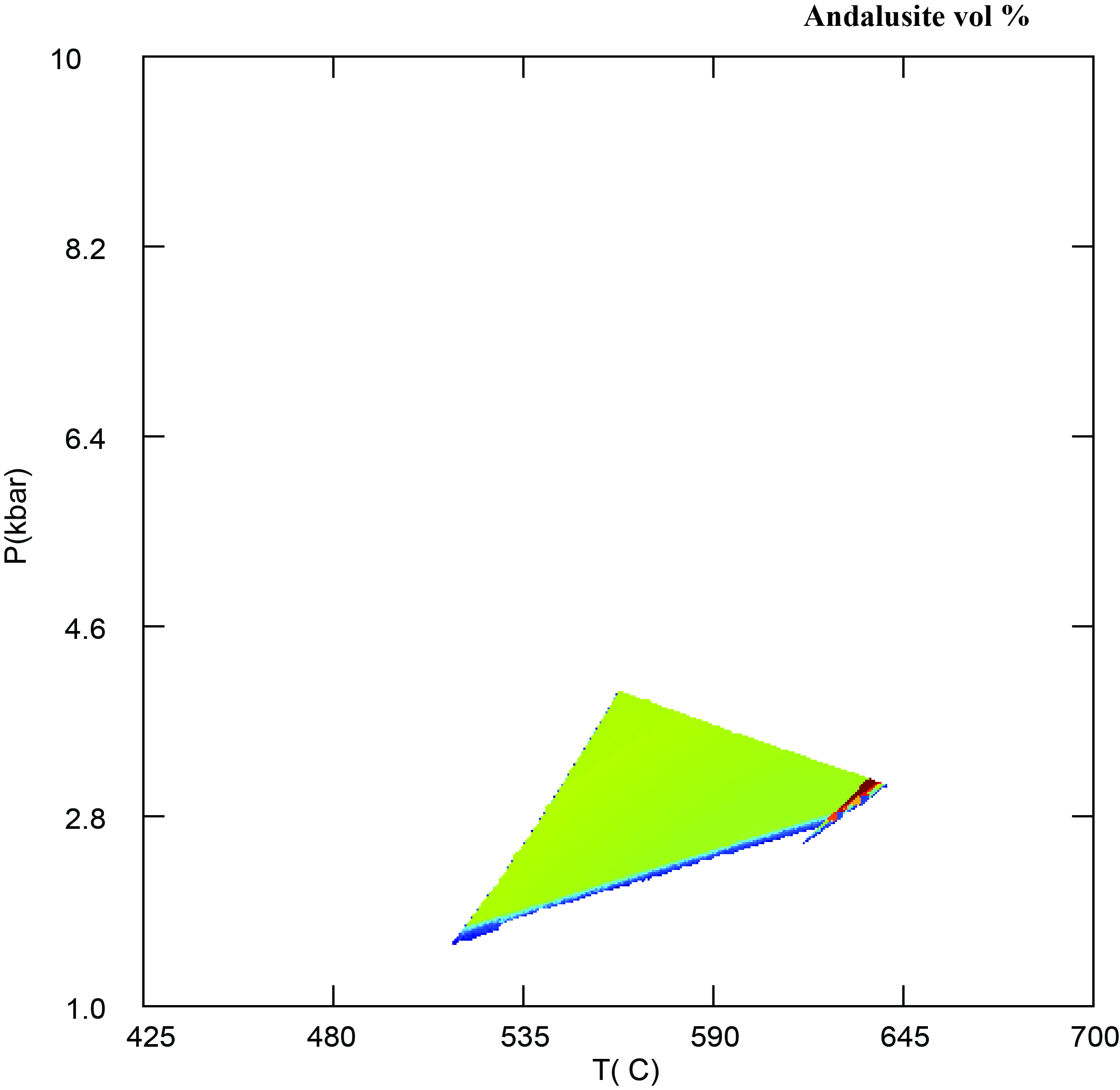

Supplement: Supplementary file 4 [file mmc4.zip › Pseudosections/Pseudosections/D472/Isomodes/Andalusite.tif]

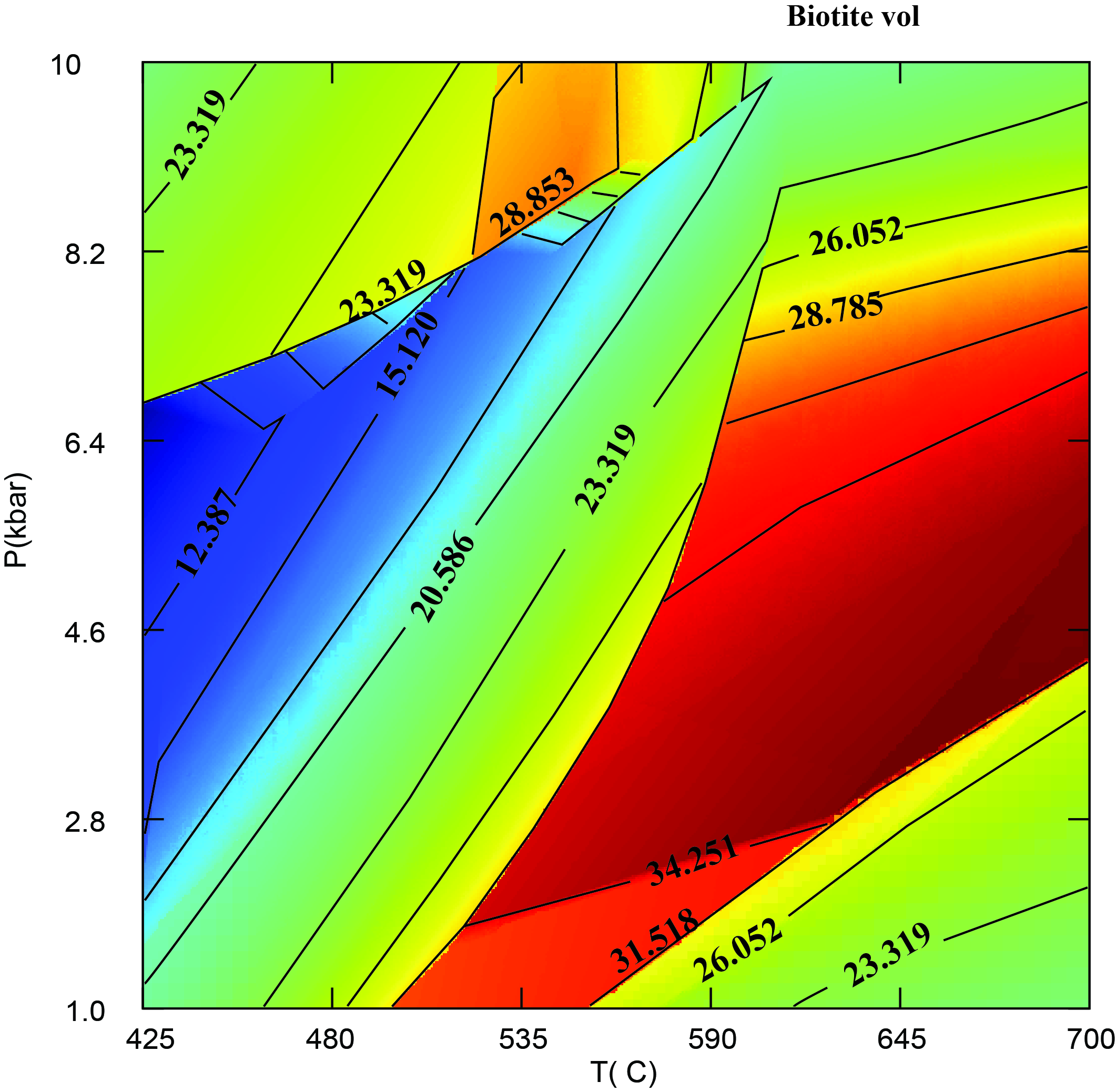

Supplement: Supplementary file 4 [file mmc4.zip › Pseudosections/Pseudosections/D472/Isomodes/Biotite vol.tif]

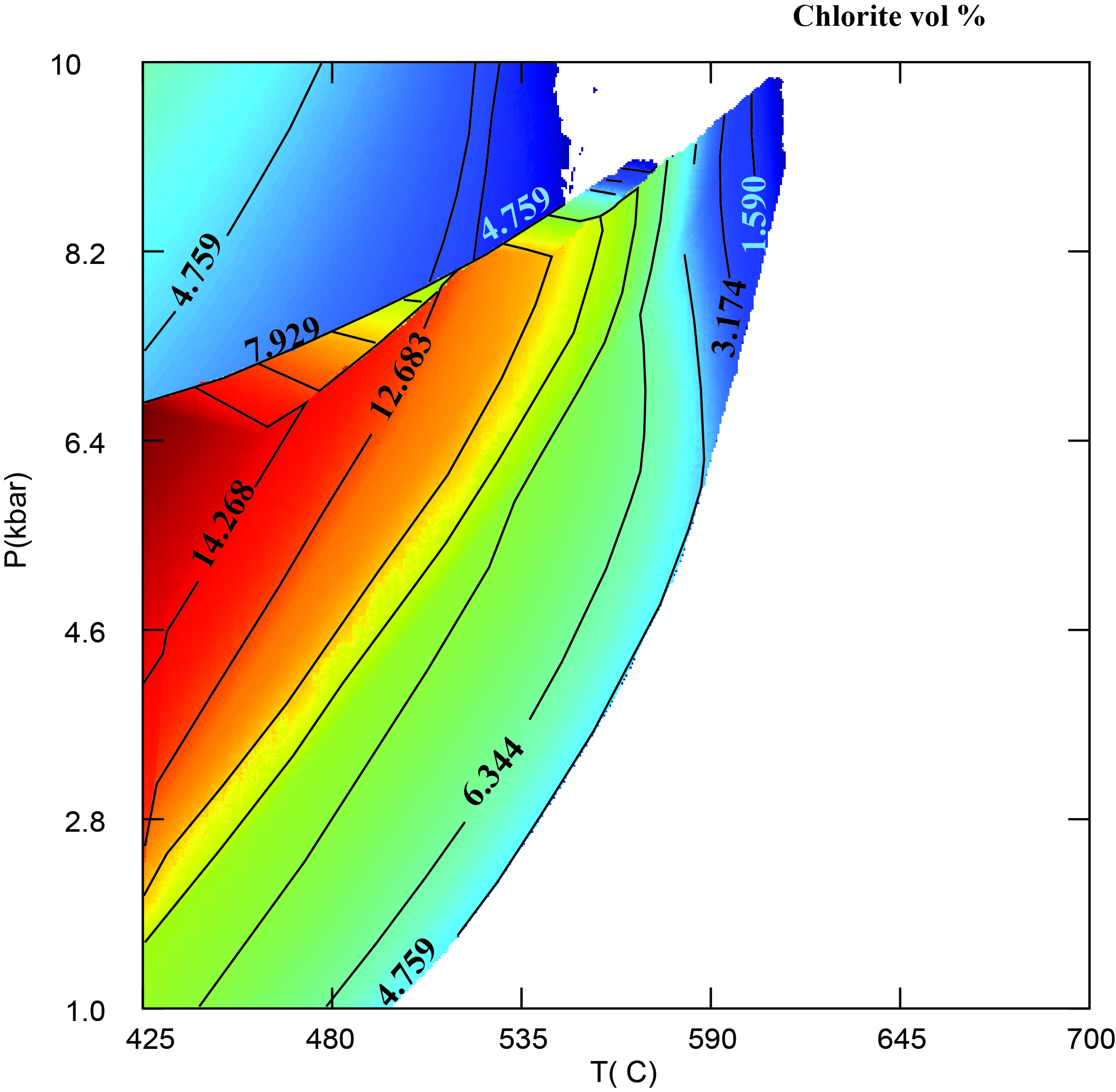

Supplement: Supplementary file 4 [file mmc4.zip › Pseudosections/Pseudosections/D472/Isomodes/Chlorite vol.tif]

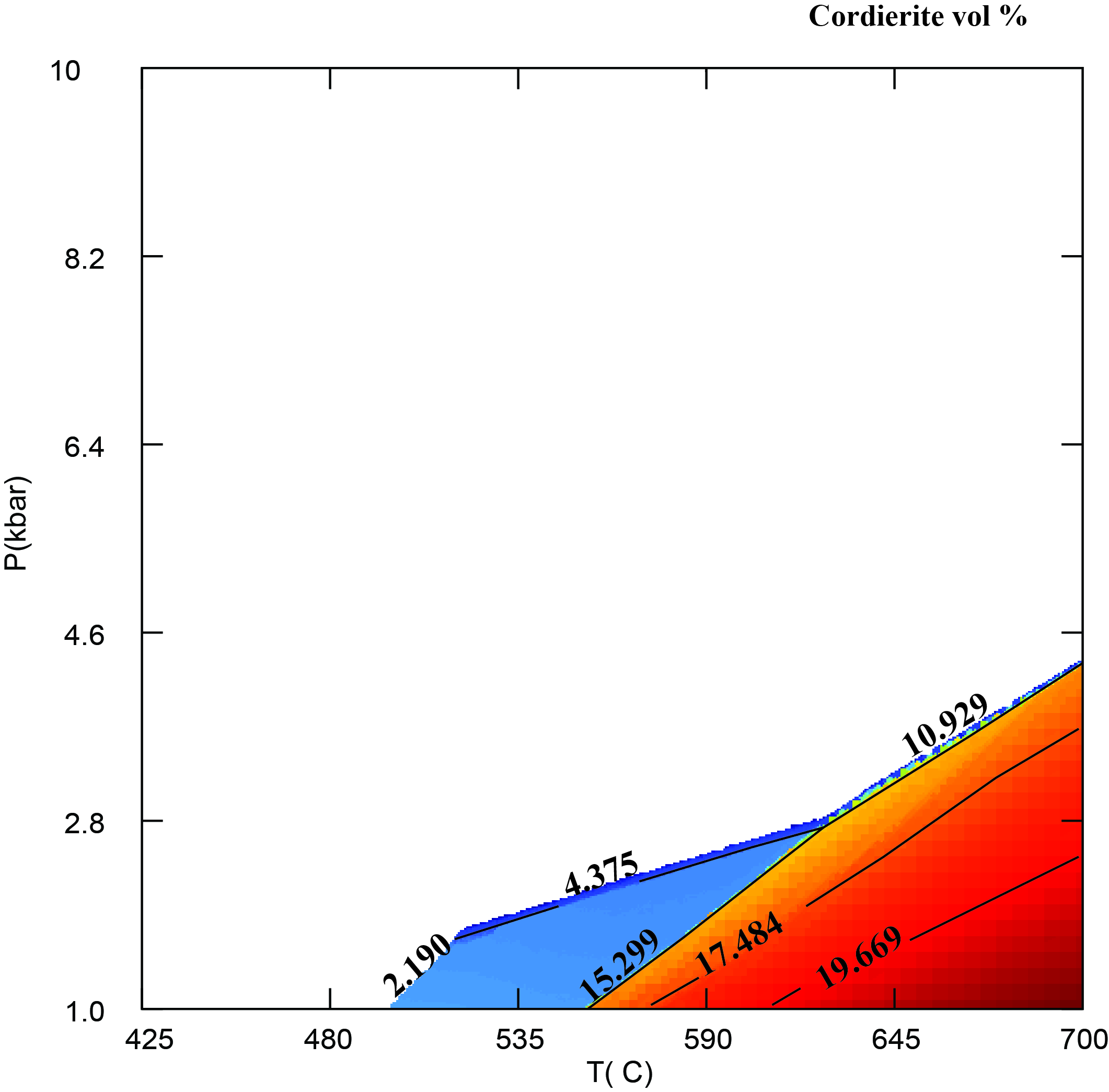

Supplement: Supplementary file 4 [file mmc4.zip › Pseudosections/Pseudosections/D472/Isomodes/Cordierite.tif]

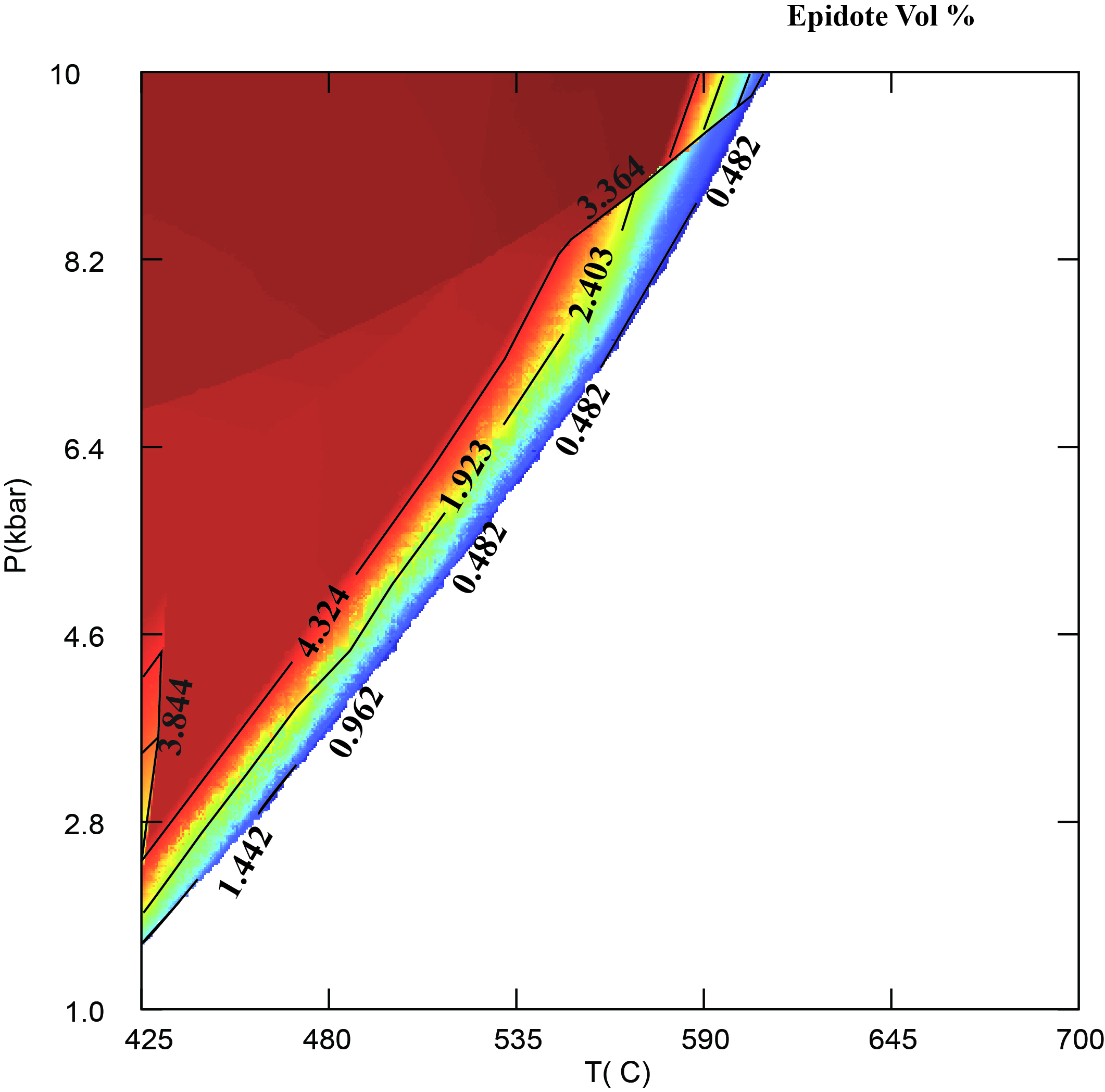

Supplement: Supplementary file 4 [file mmc4.zip › Pseudosections/Pseudosections/D472/Isomodes/Epidote.tif]

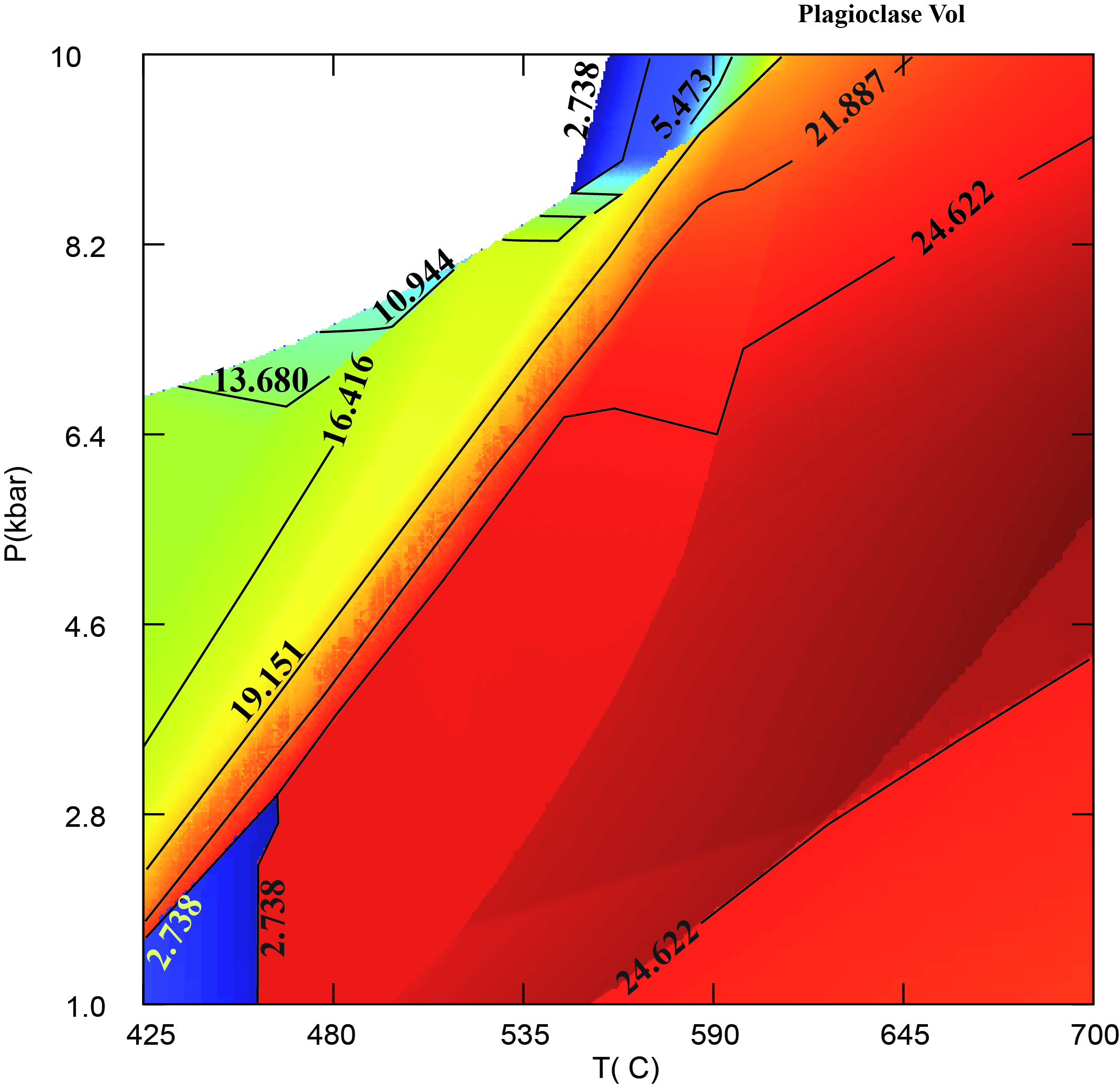

Supplement: Supplementary file 4 [file mmc4.zip › Pseudosections/Pseudosections/D472/Isomodes/feldspar.tif]

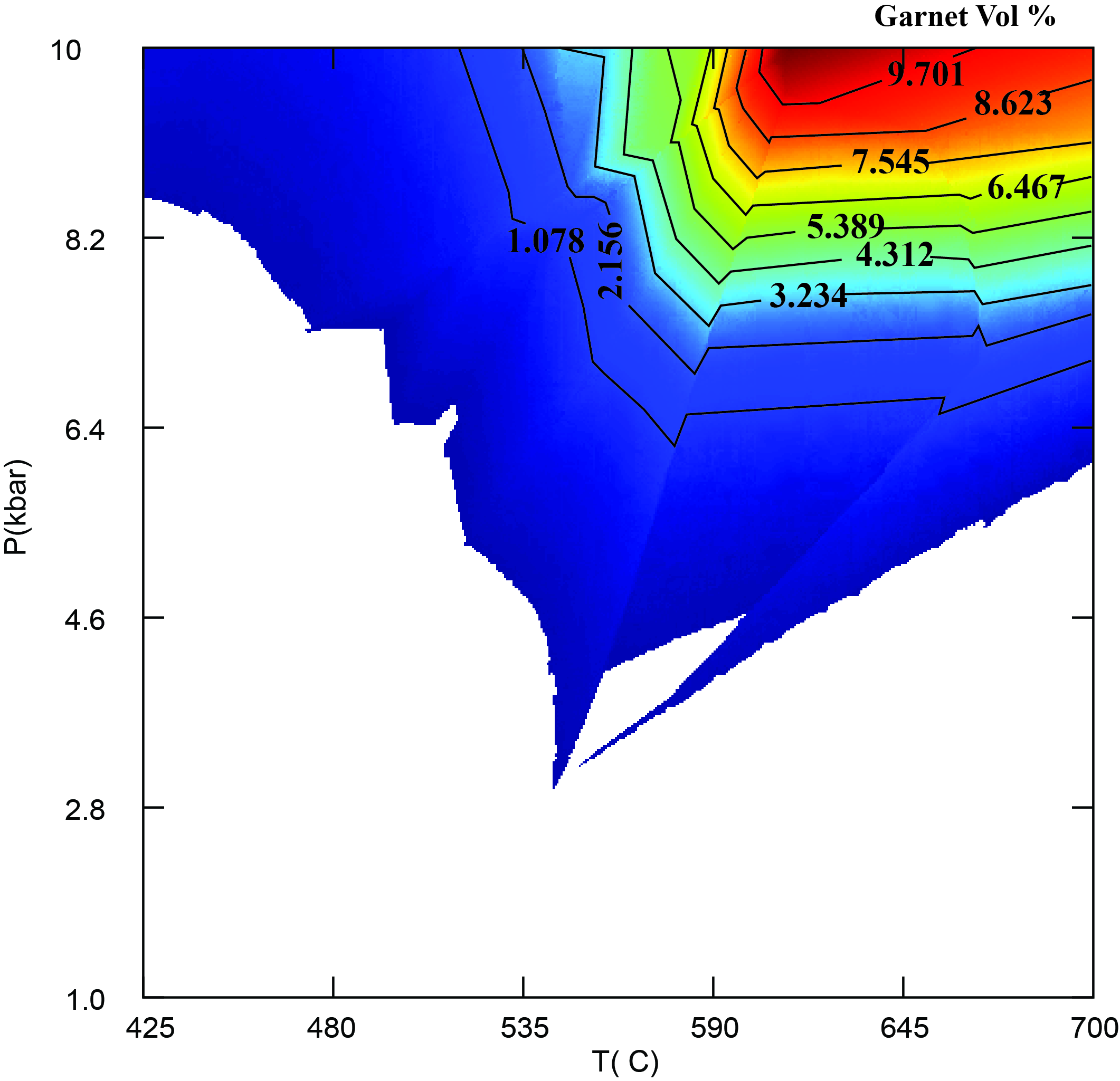

Supplement: Supplementary file 4 [file mmc4.zip › Pseudosections/Pseudosections/D472/Isomodes/Garnet.tif]

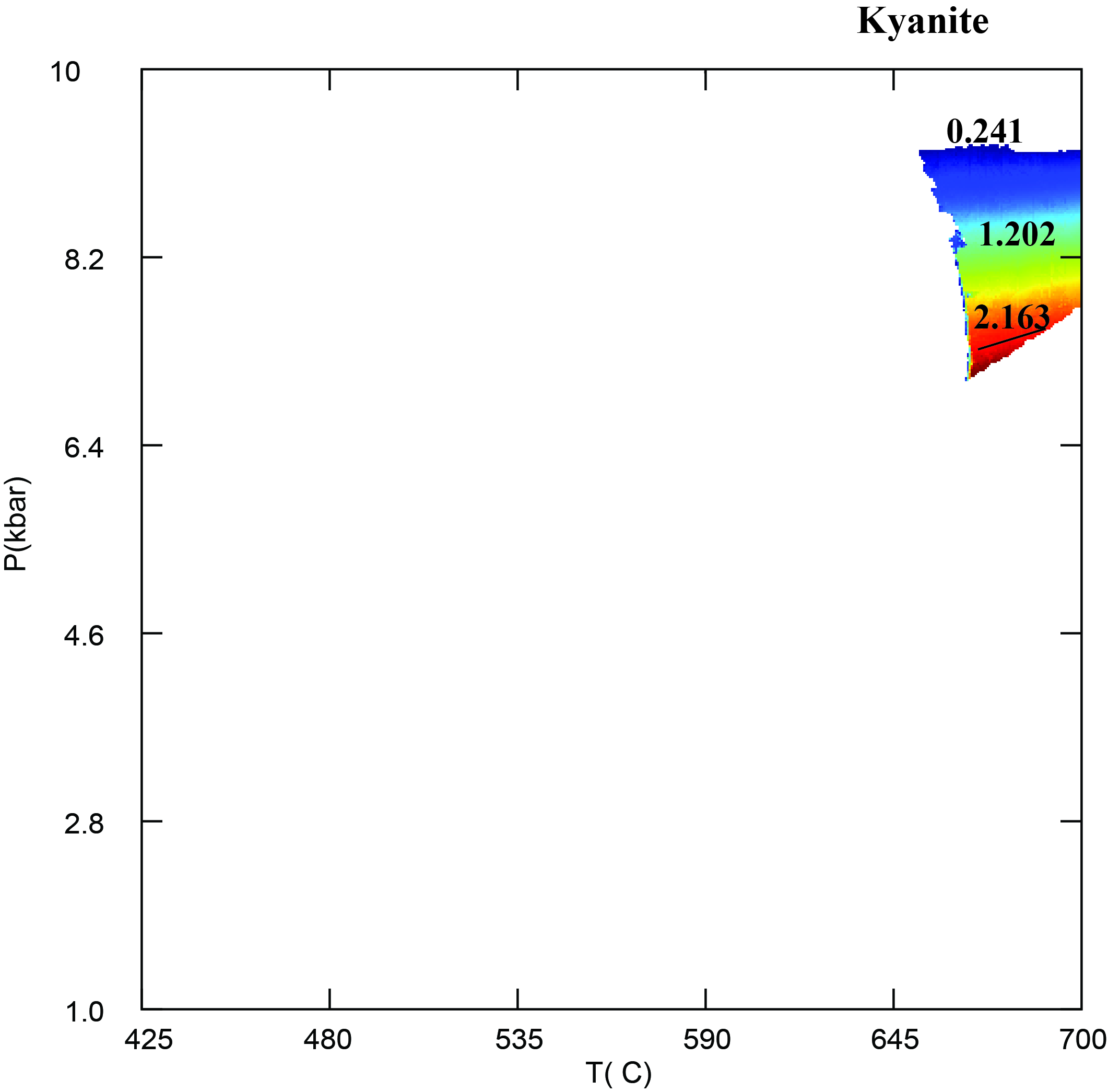

Supplement: Supplementary file 4 [file mmc4.zip › Pseudosections/Pseudosections/D472/Isomodes/Ky.tif]

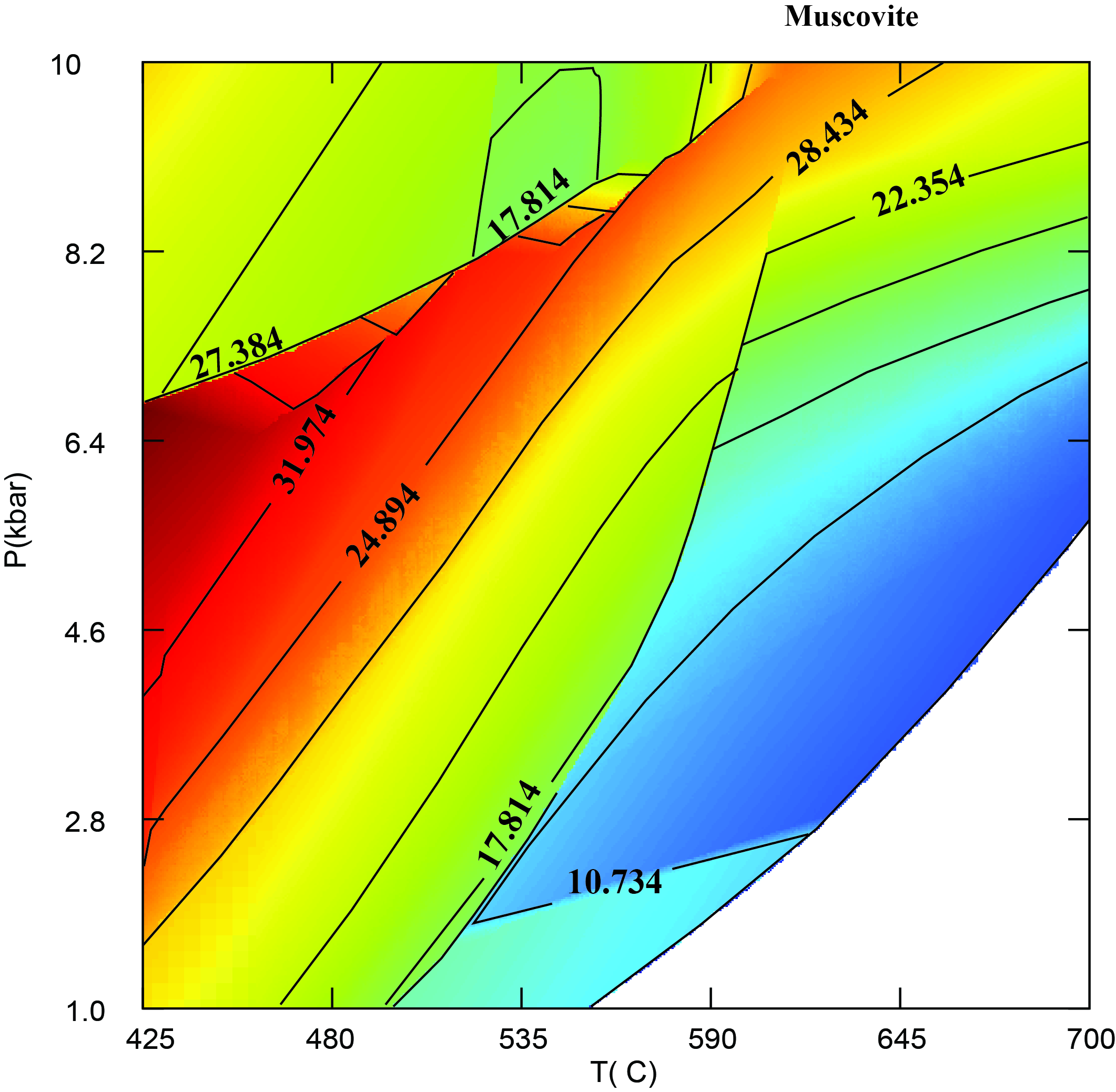

Supplement: Supplementary file 4 [file mmc4.zip › Pseudosections/Pseudosections/D472/Isomodes/Muscovite.tif]

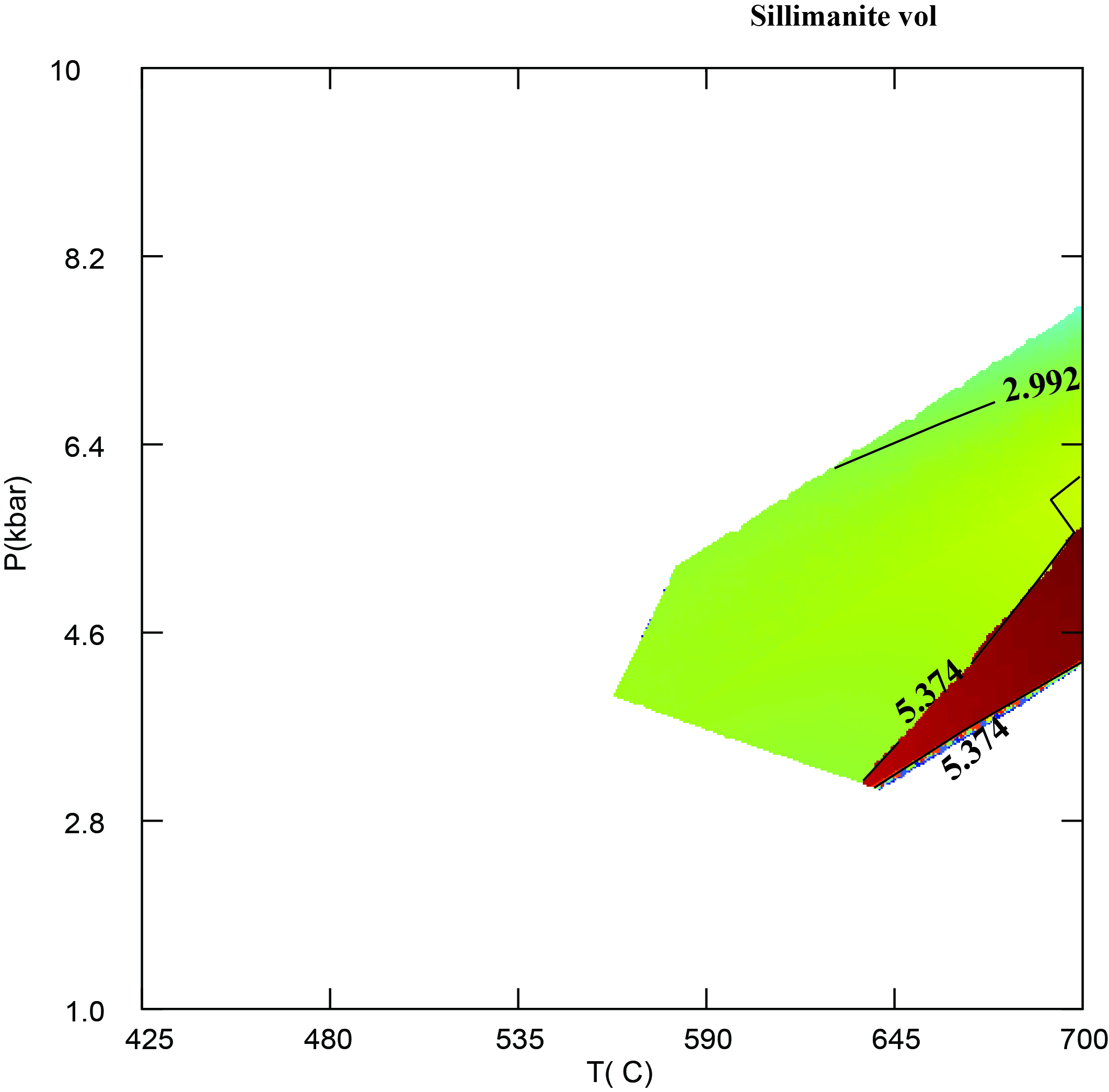

Supplement: Supplementary file 4 [file mmc4.zip › Pseudosections/Pseudosections/D472/Isomodes/Sill vol.tif]

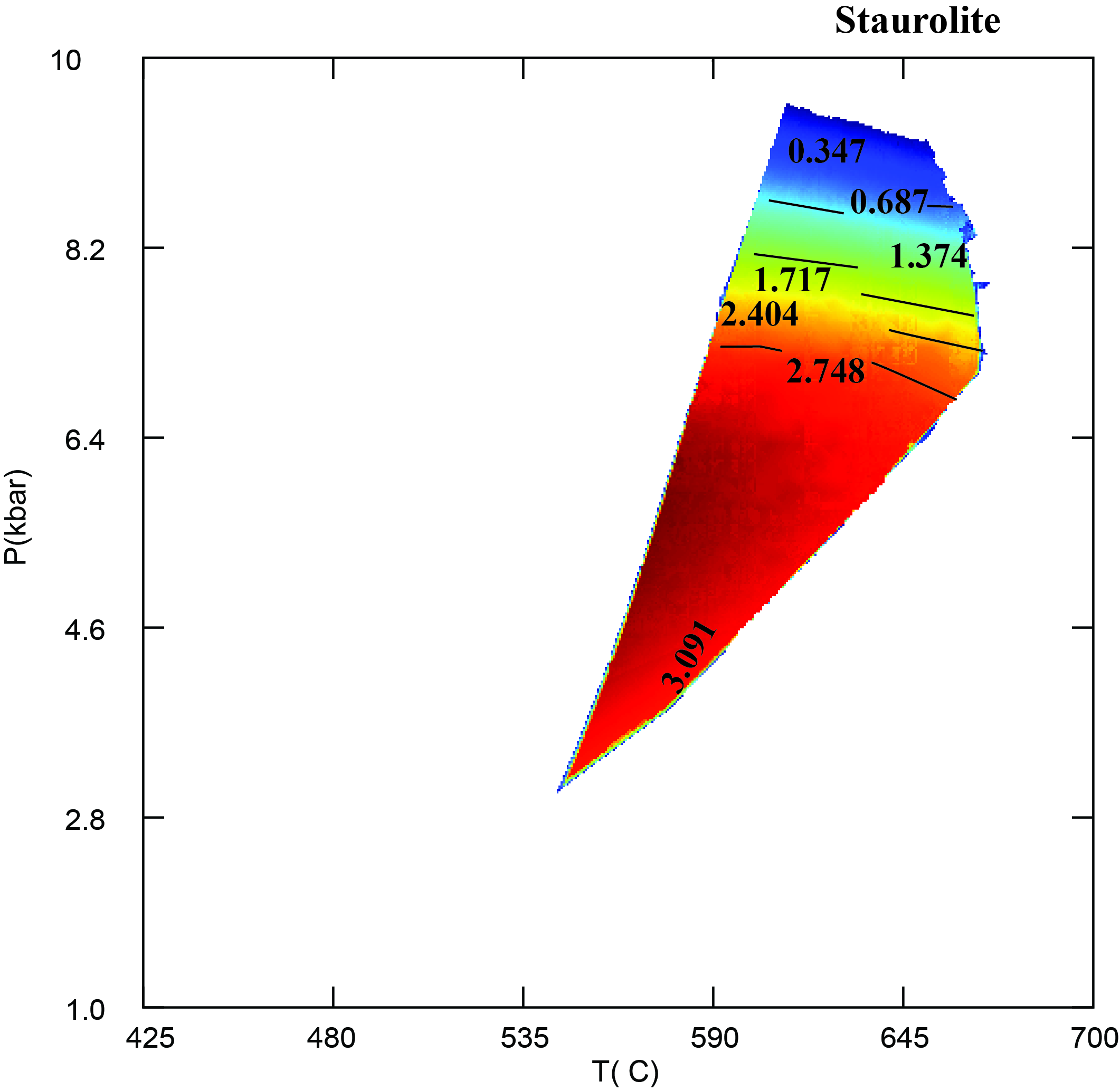

Supplement: Supplementary file 4 [file mmc4.zip › Pseudosections/Pseudosections/D472/Isomodes/Staurolite vol.tif]

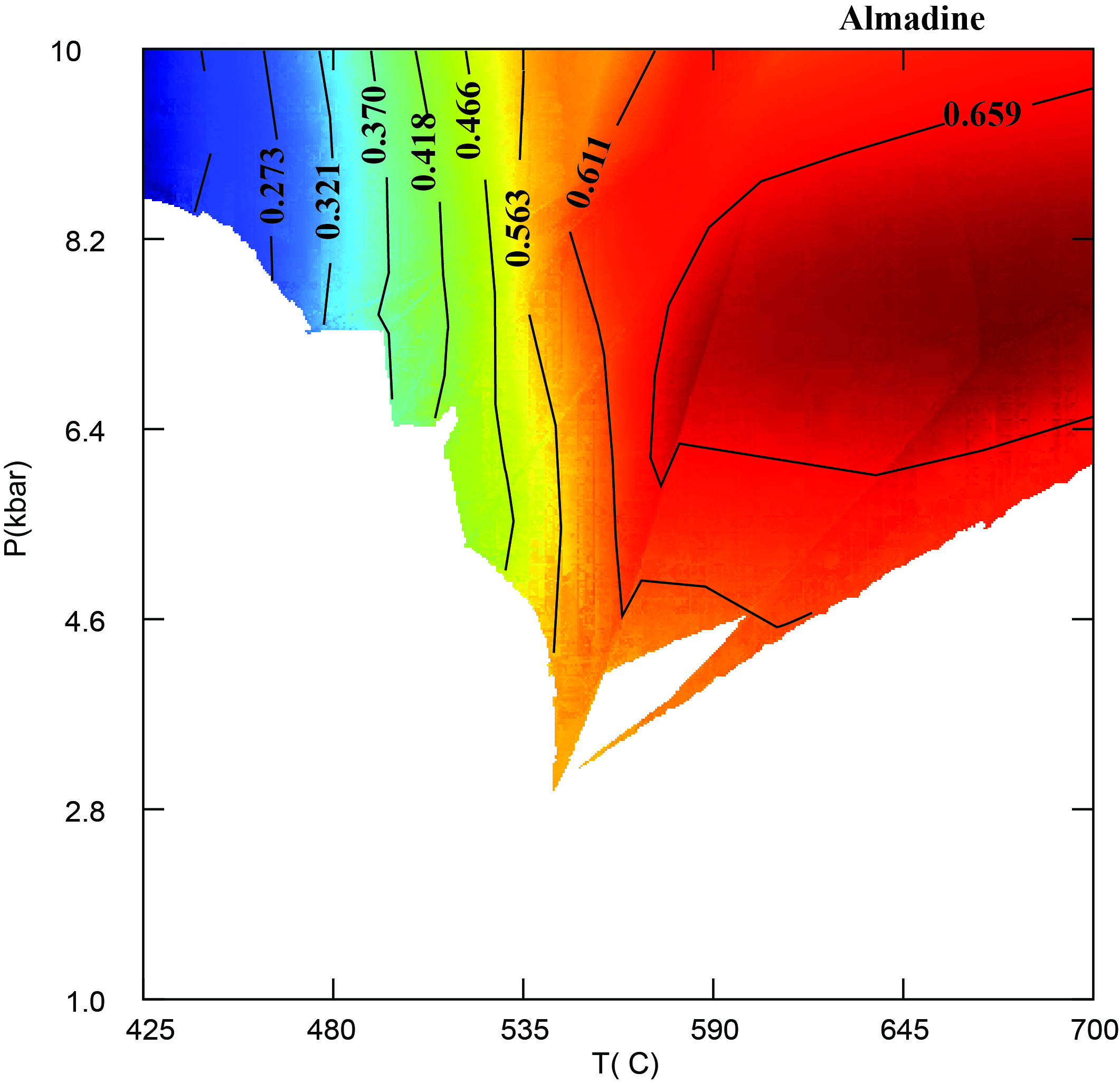

Supplement: Supplementary file 4 [file mmc4.zip › Pseudosections/Pseudosections/D472/Isopleths/Alm.tif]

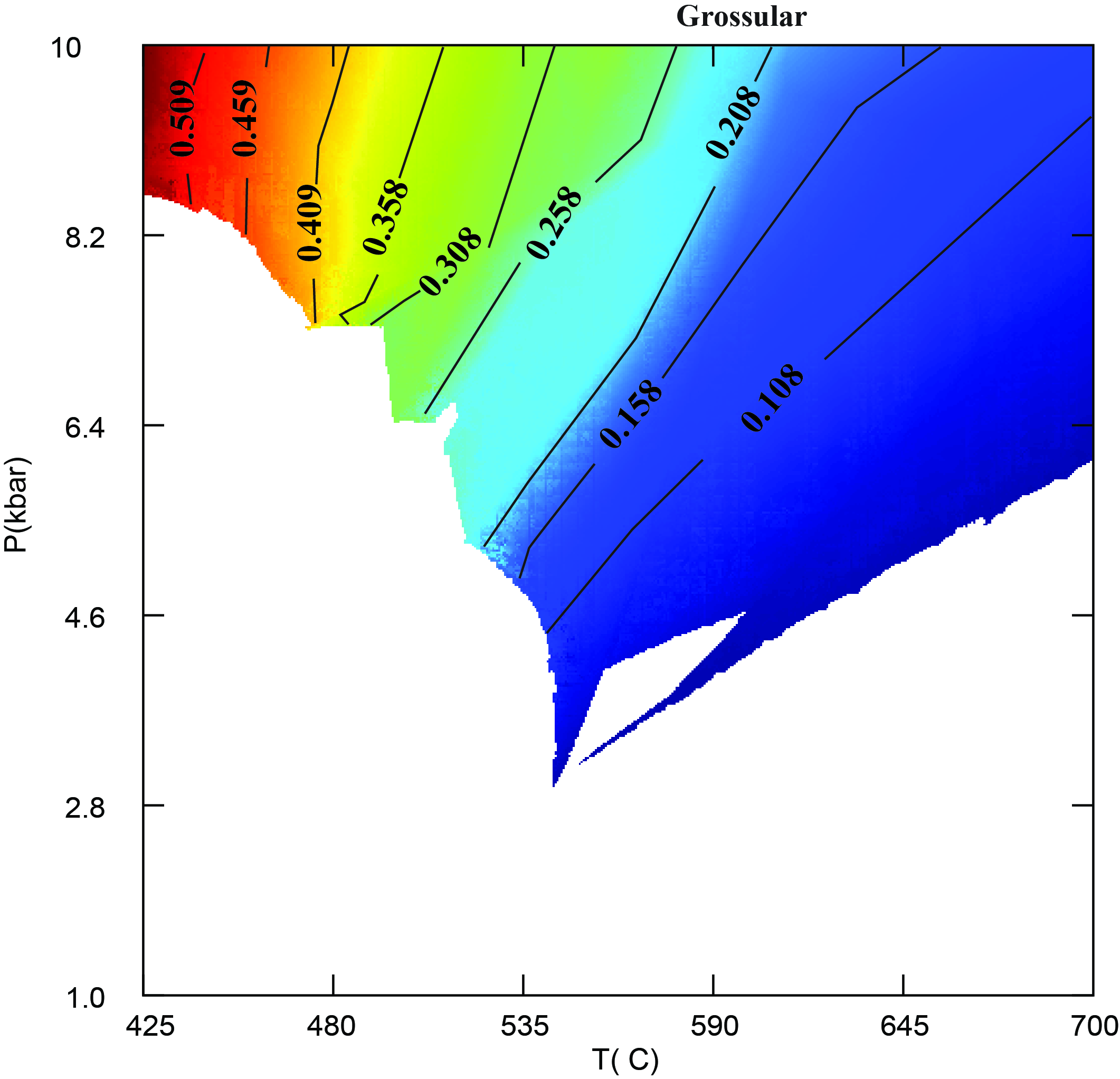

Supplement: Supplementary file 4 [file mmc4.zip › Pseudosections/Pseudosections/D472/Isopleths/Grss.tif]

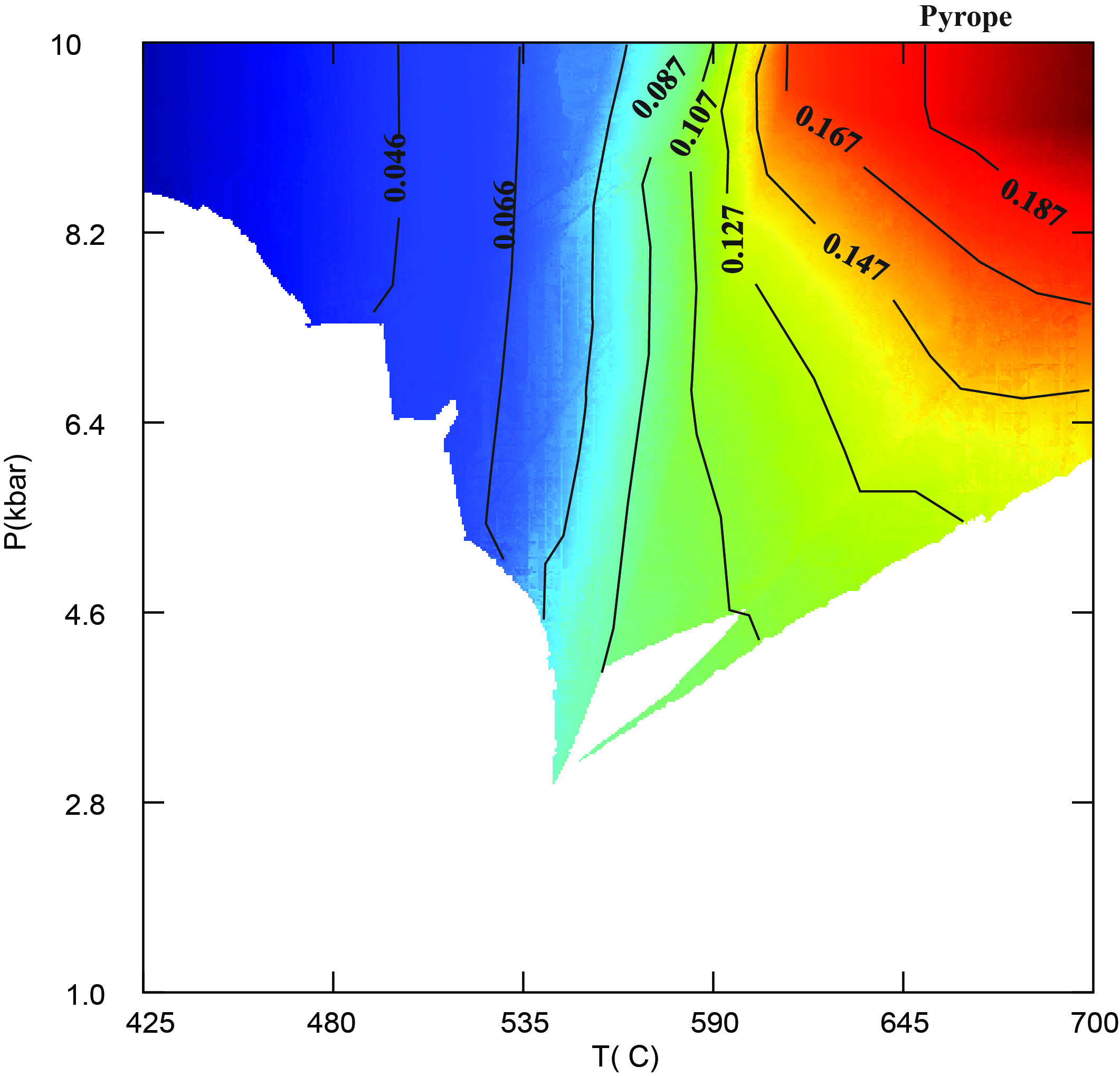

Supplement: Supplementary file 4 [file mmc4.zip › Pseudosections/Pseudosections/D472/Isopleths/Pyr.tif]

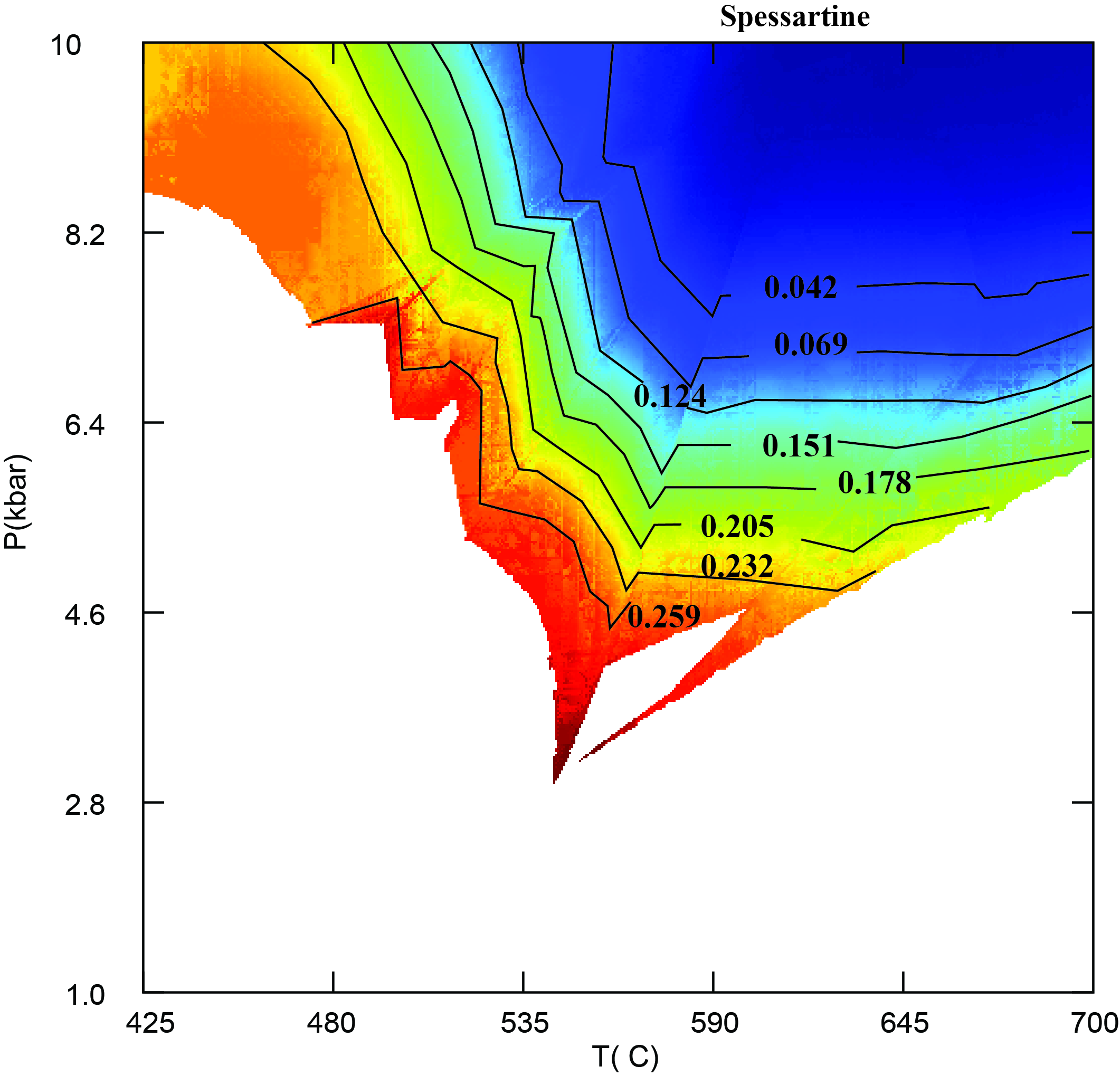

Supplement: Supplementary file 4 [file mmc4.zip › Pseudosections/Pseudosections/D472/Isopleths/Spessartine.tif]

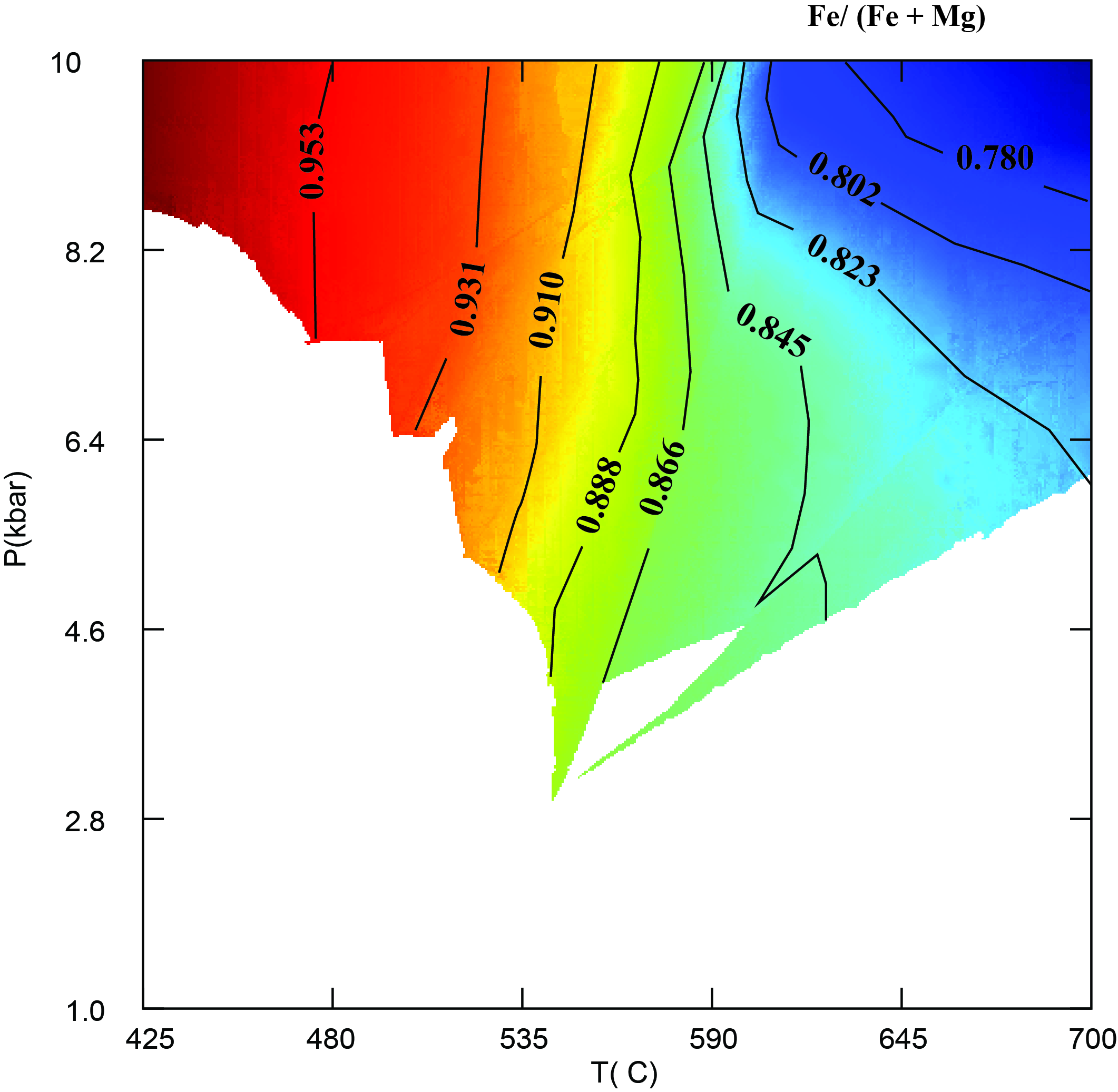

Supplement: Supplementary file 4 [file mmc4.zip › Pseudosections/Pseudosections/D472/Isopleths/XFe Garnet.tif]

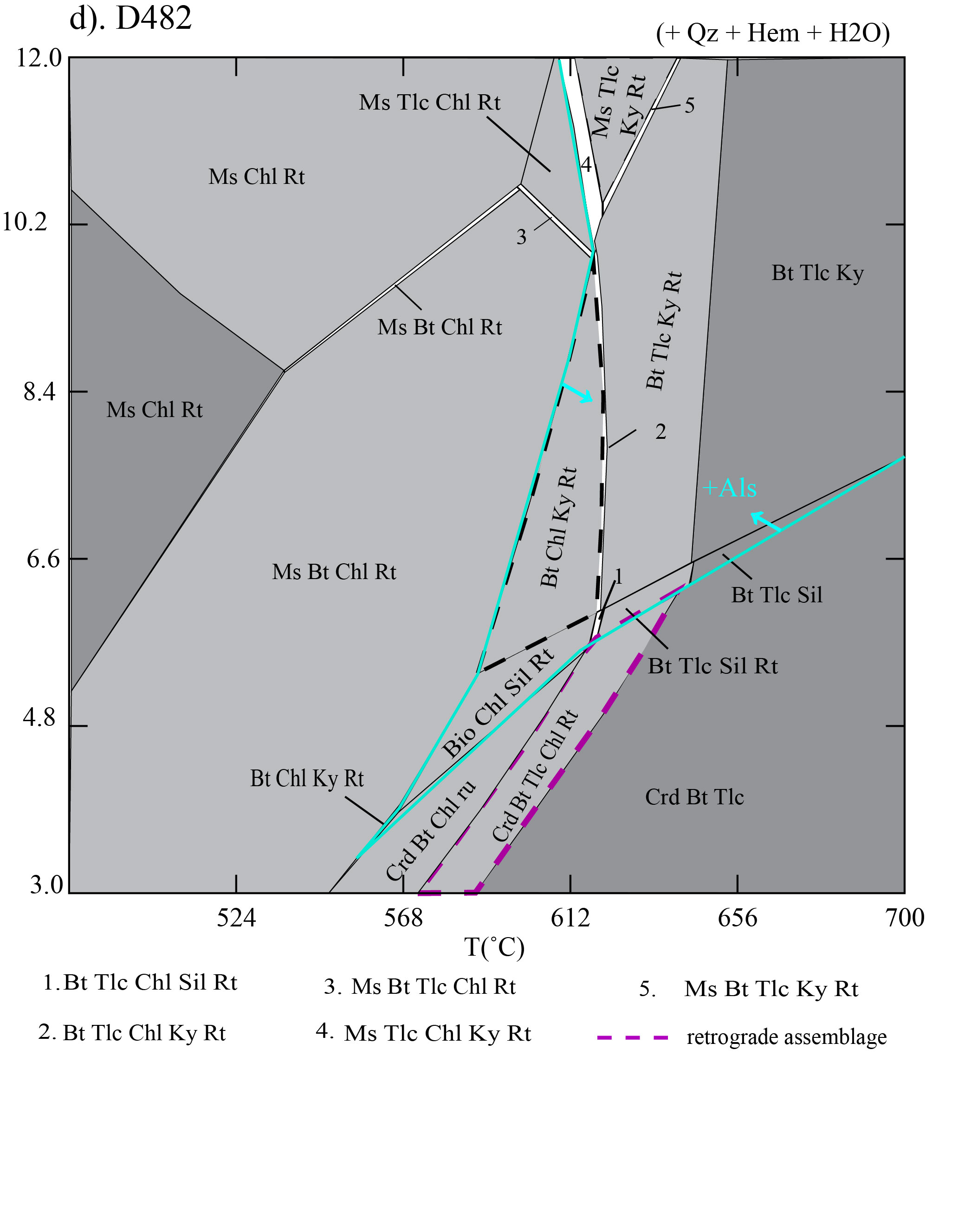

Supplement: Supplementary file 4 [file mmc4.zip › Pseudosections/Pseudosections/D482/D482.jpg]

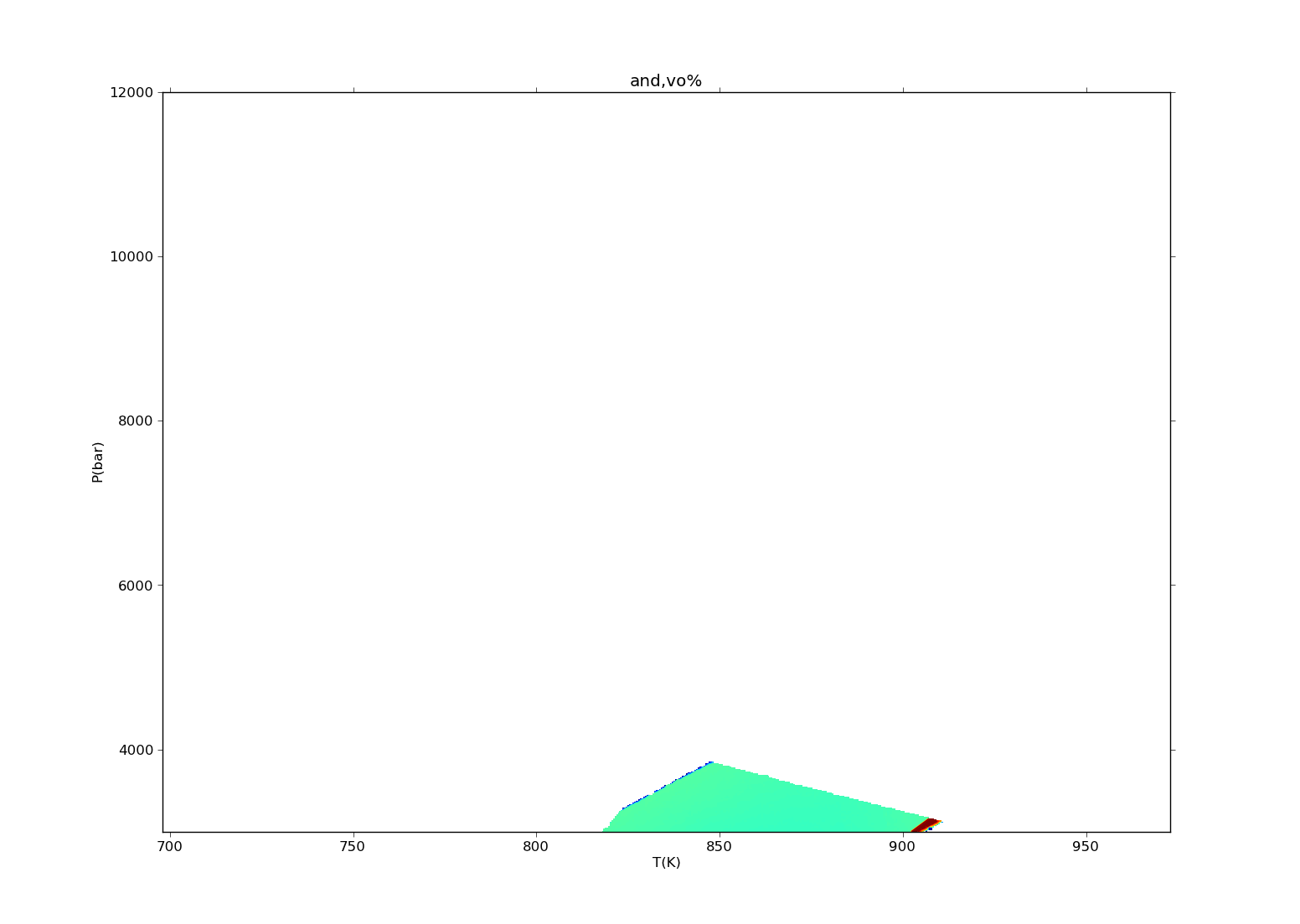

Supplement: Supplementary file 4 [file mmc4.zip › Pseudosections/Pseudosections/D490/isomodes/And_vol%.png]

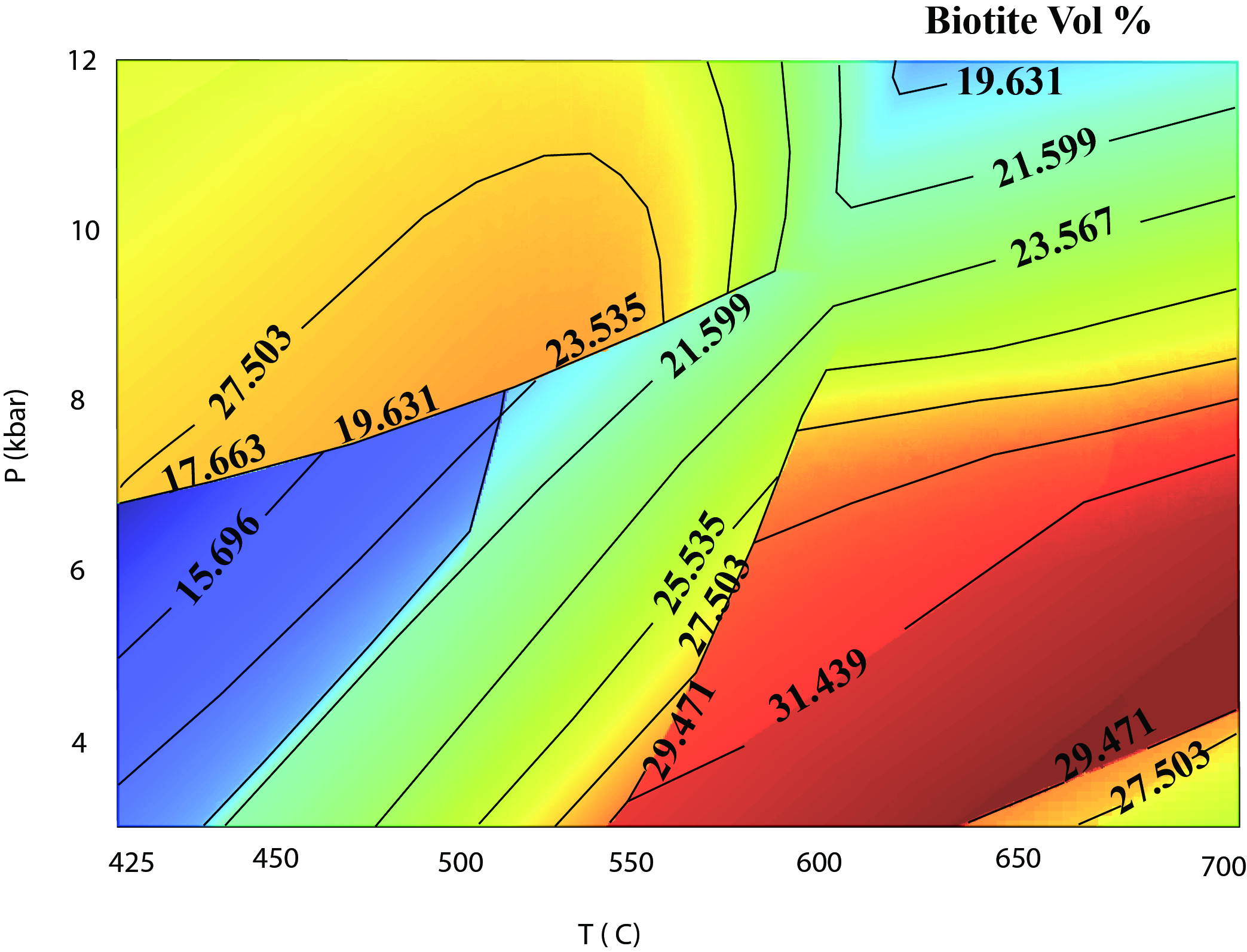

Supplement: Supplementary file 4 [file mmc4.zip › Pseudosections/Pseudosections/D490/isomodes/Biotite edited.tif]

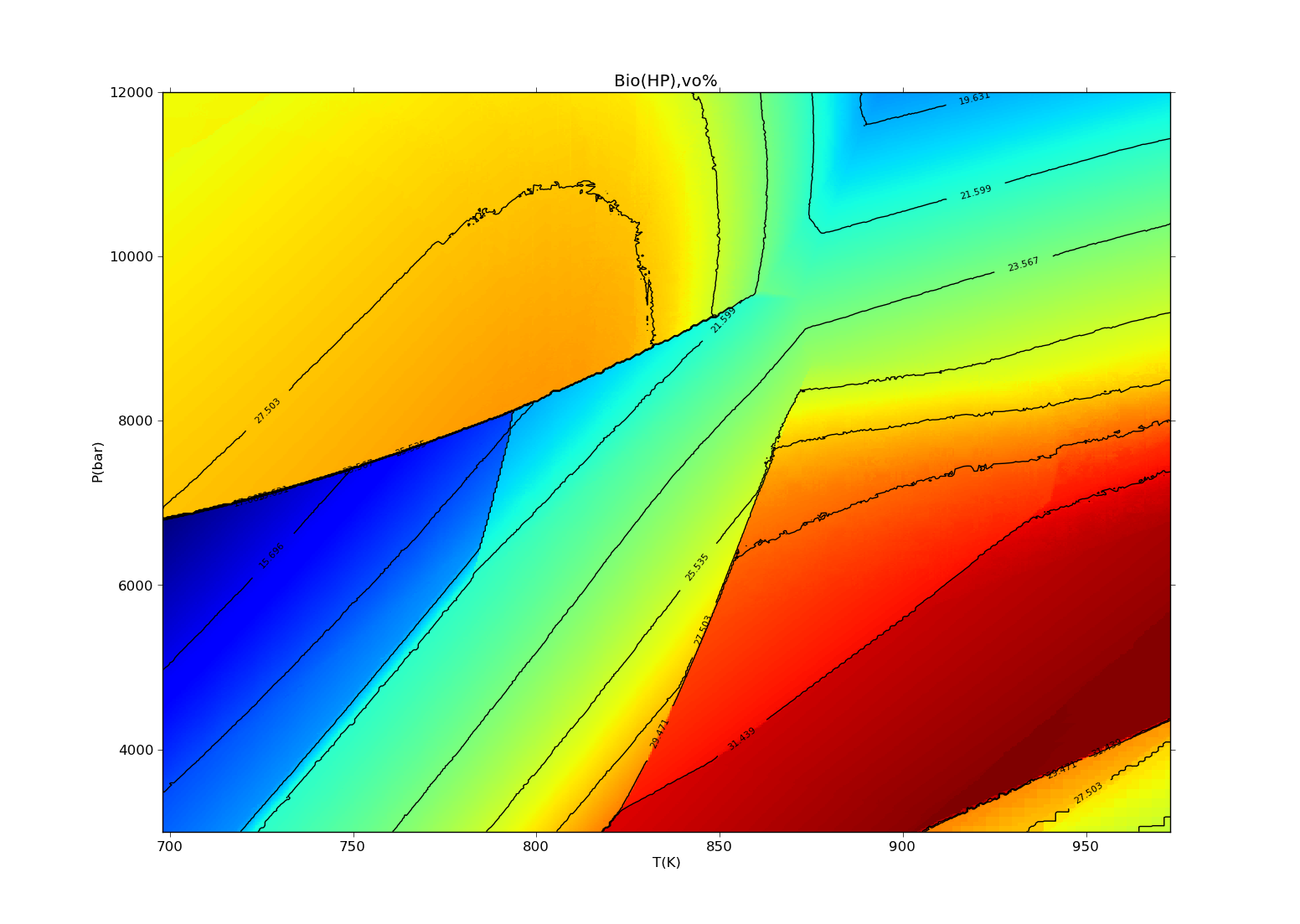

Supplement: Supplementary file 4 [file mmc4.zip › Pseudosections/Pseudosections/D490/isomodes/Biotite.png]

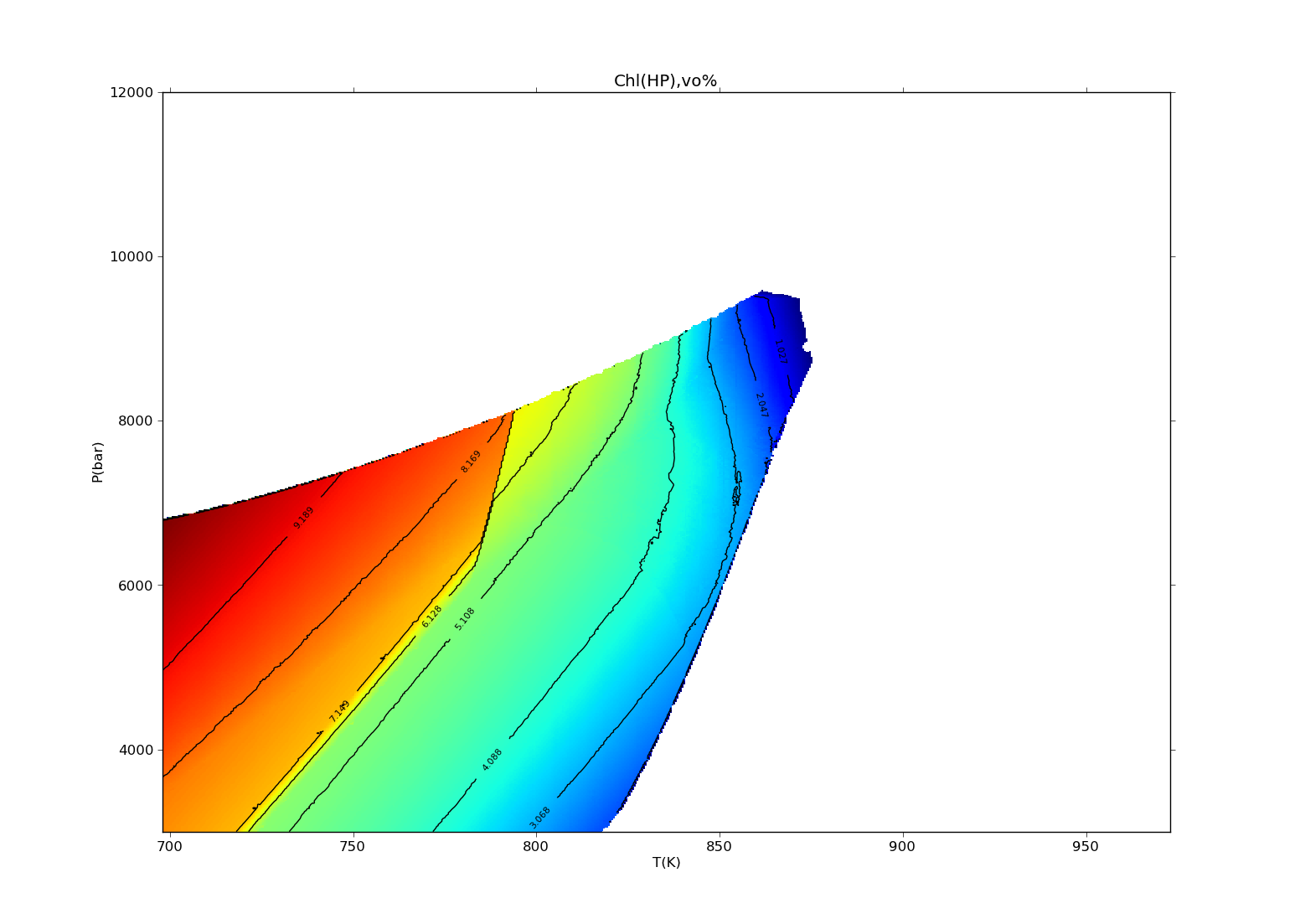

Supplement: Supplementary file 4 [file mmc4.zip › Pseudosections/Pseudosections/D490/isomodes/Chl.png]

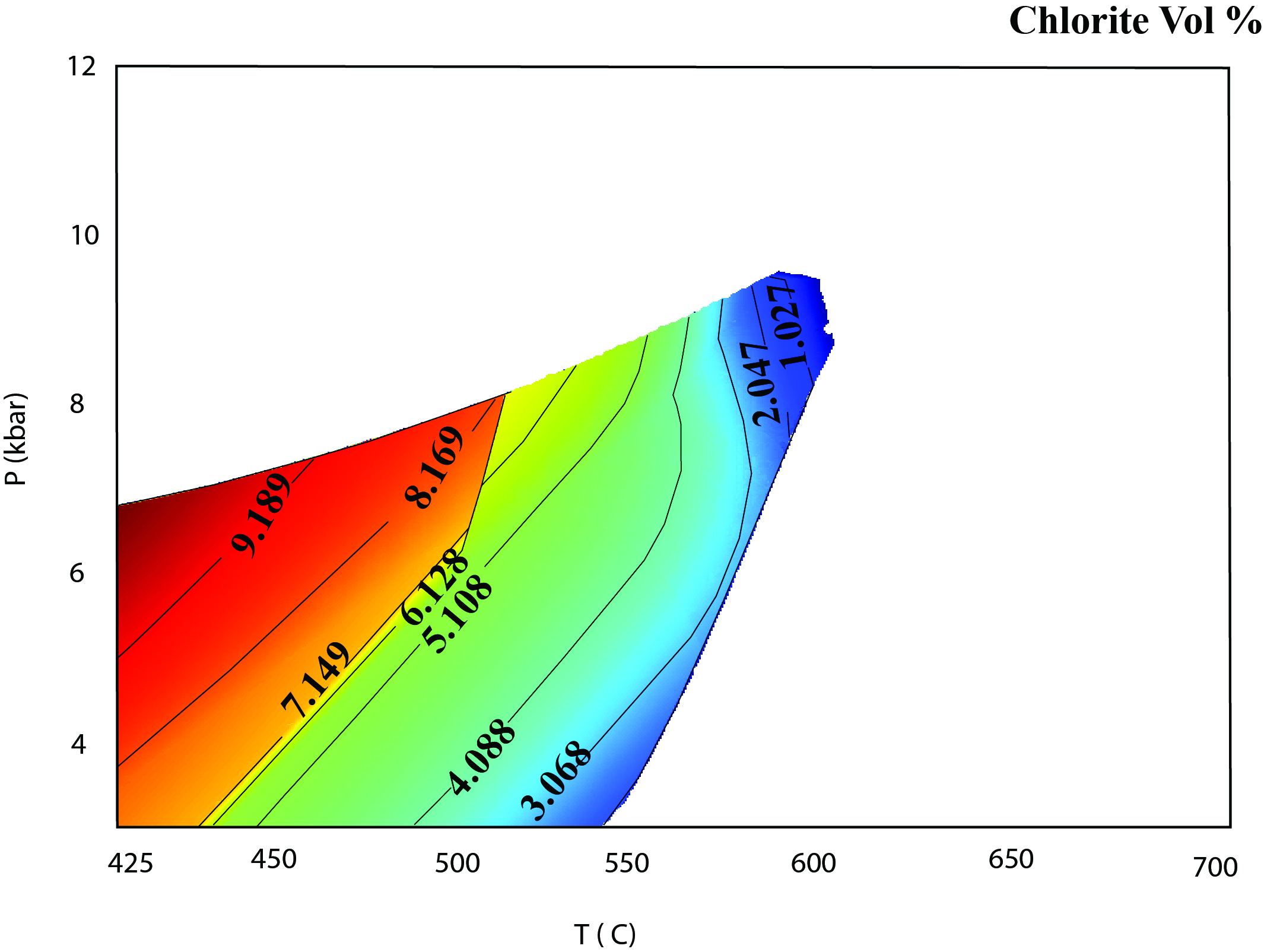

Supplement: Supplementary file 4 [file mmc4.zip › Pseudosections/Pseudosections/D490/isomodes/Chlorite edited.tif]

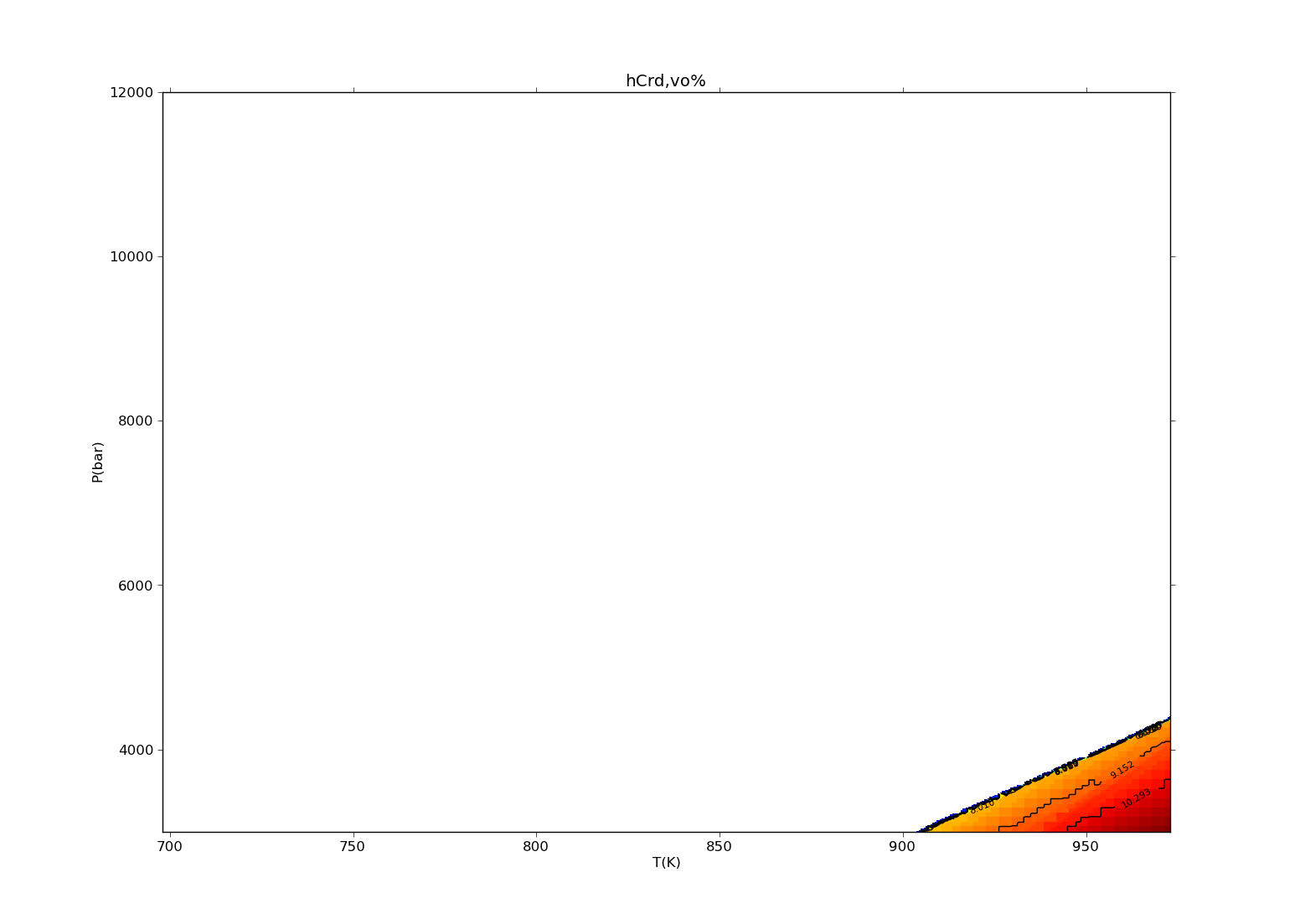

Supplement: Supplementary file 4 [file mmc4.zip › Pseudosections/Pseudosections/D490/isomodes/Crd.png]

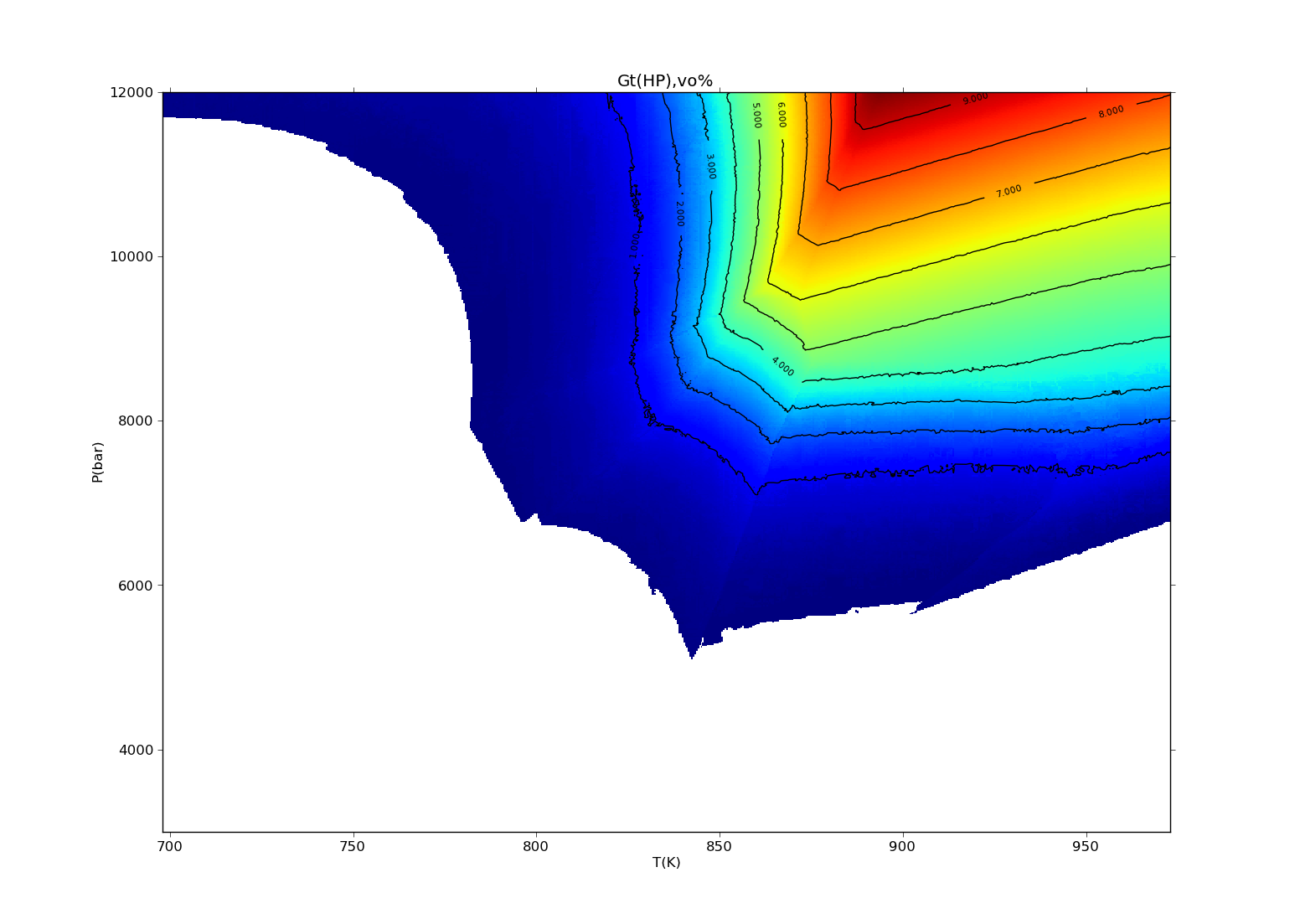

Supplement: Supplementary file 4 [file mmc4.zip › Pseudosections/Pseudosections/D490/isomodes/Gt.png]

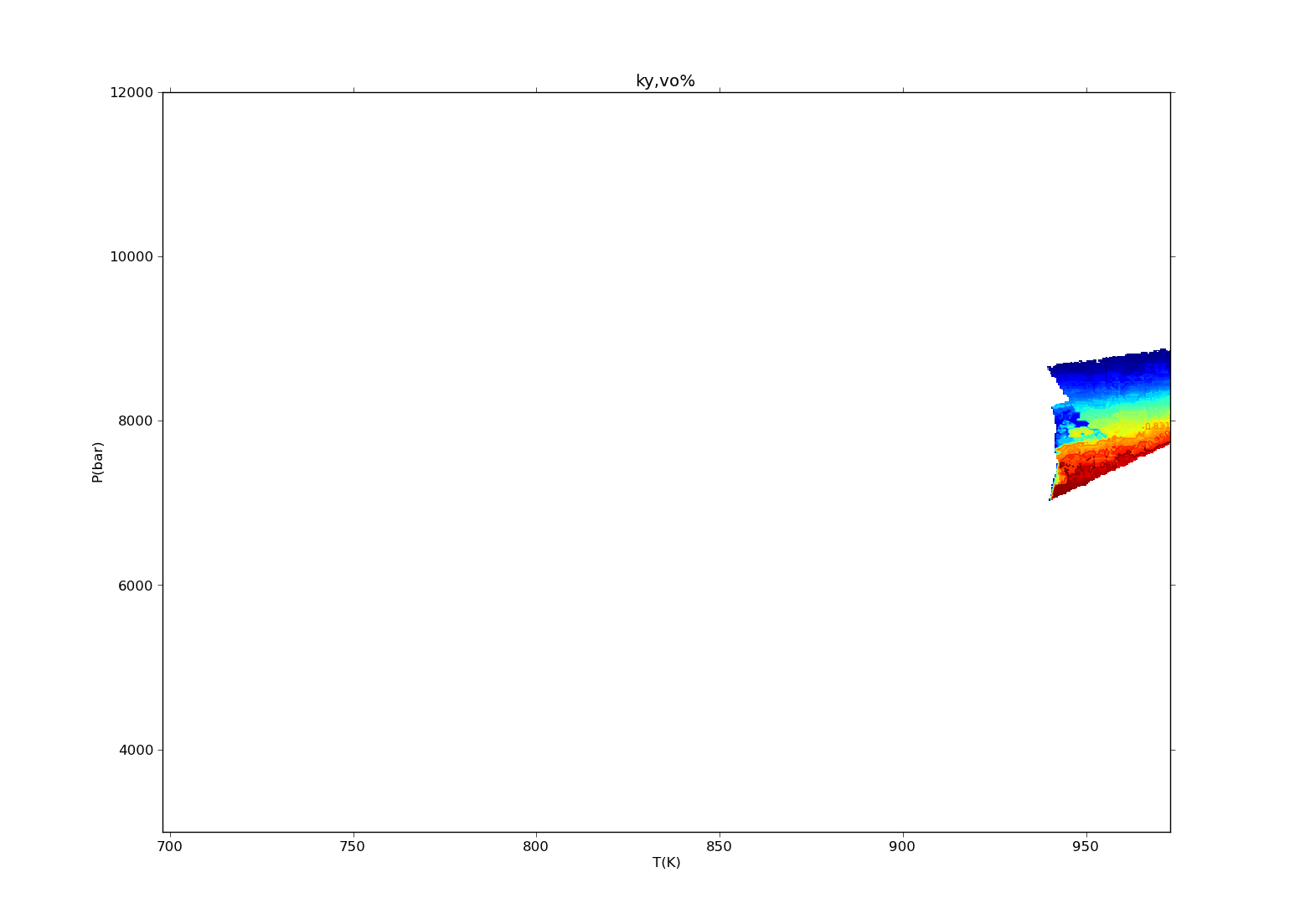

Supplement: Supplementary file 4 [file mmc4.zip › Pseudosections/Pseudosections/D490/isomodes/Ky_vol%.png]

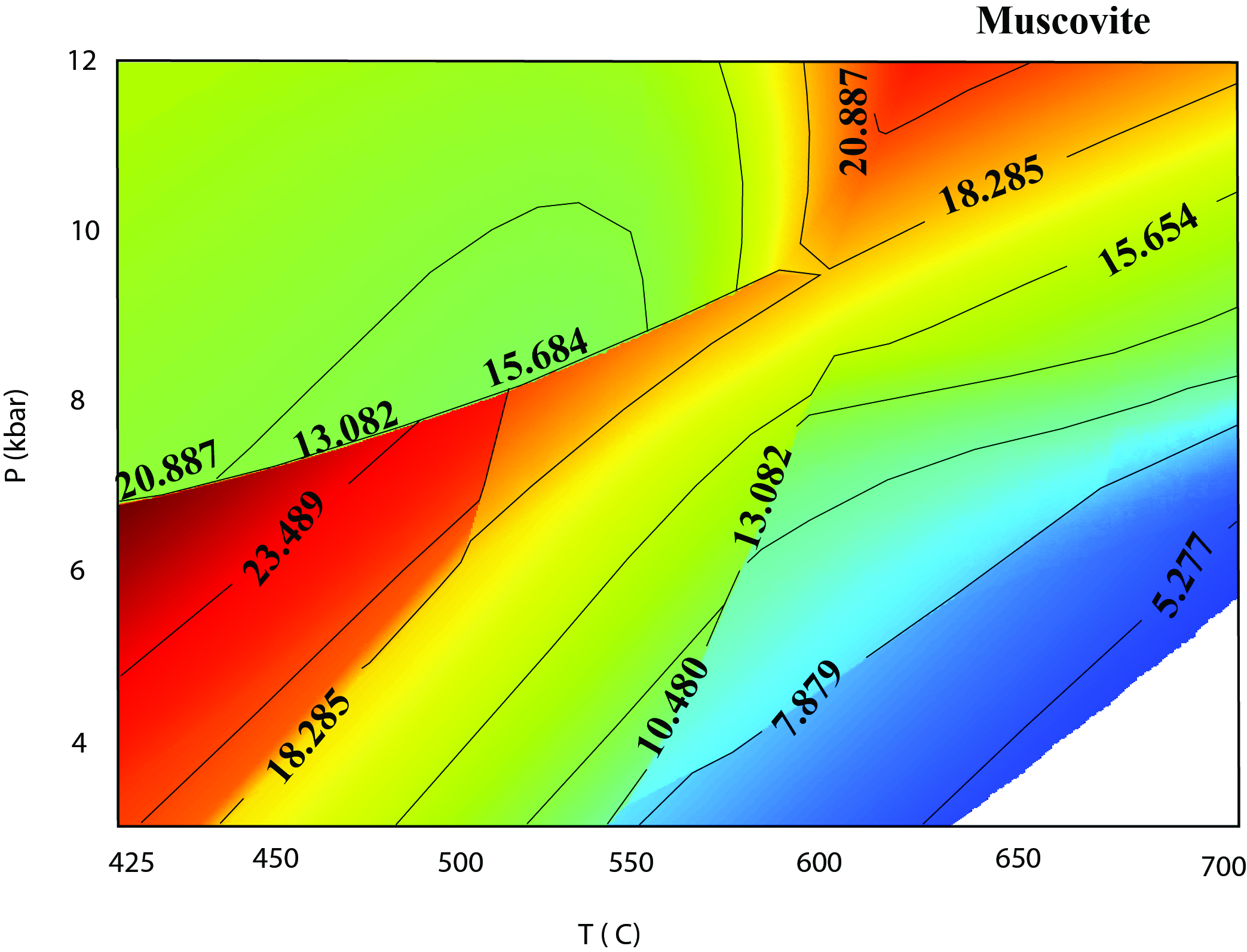

Supplement: Supplementary file 4 [file mmc4.zip › Pseudosections/Pseudosections/D490/isomodes/Muscovite edited.tif]

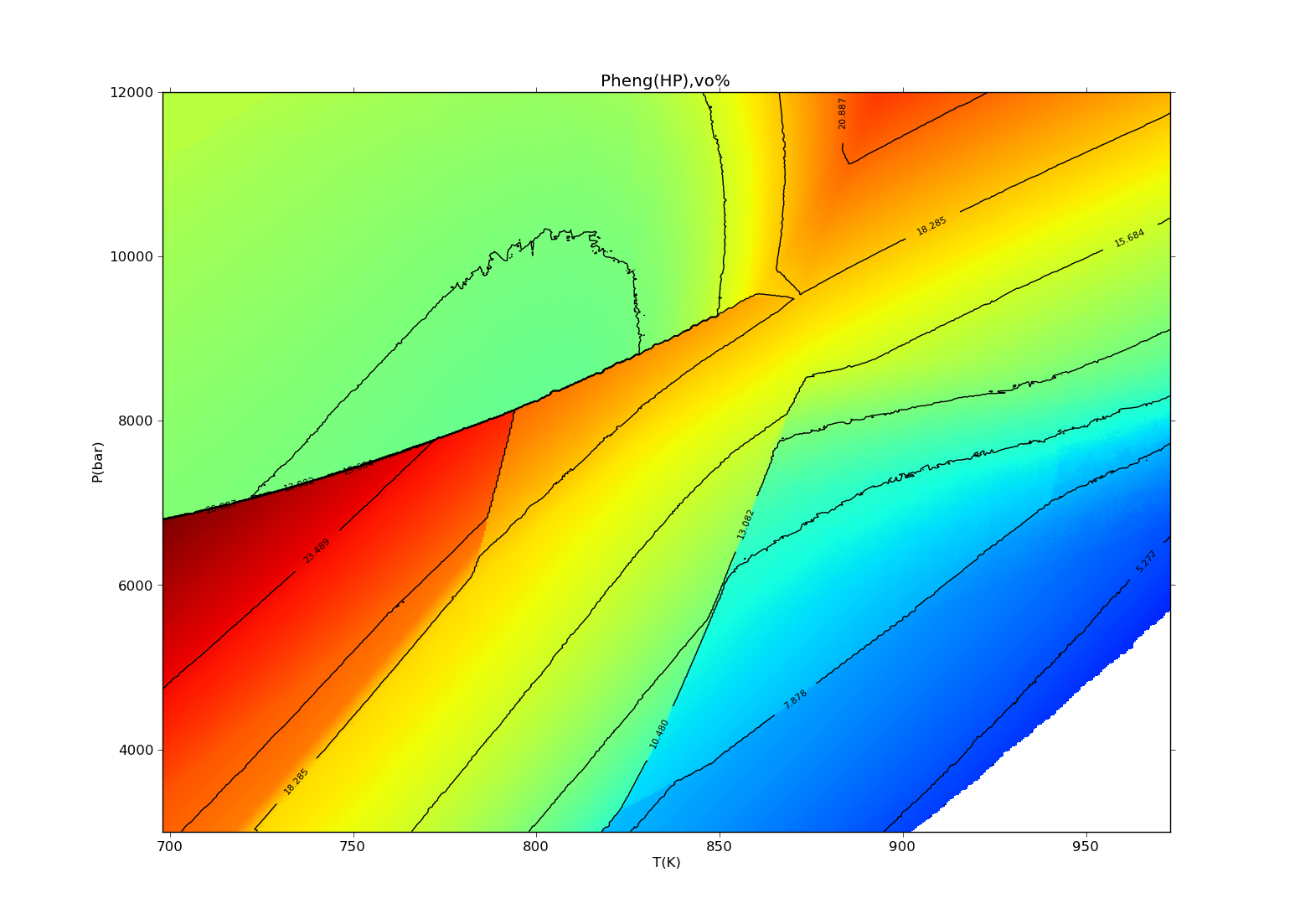

Supplement: Supplementary file 4 [file mmc4.zip › Pseudosections/Pseudosections/D490/isomodes/Muscovite.png]

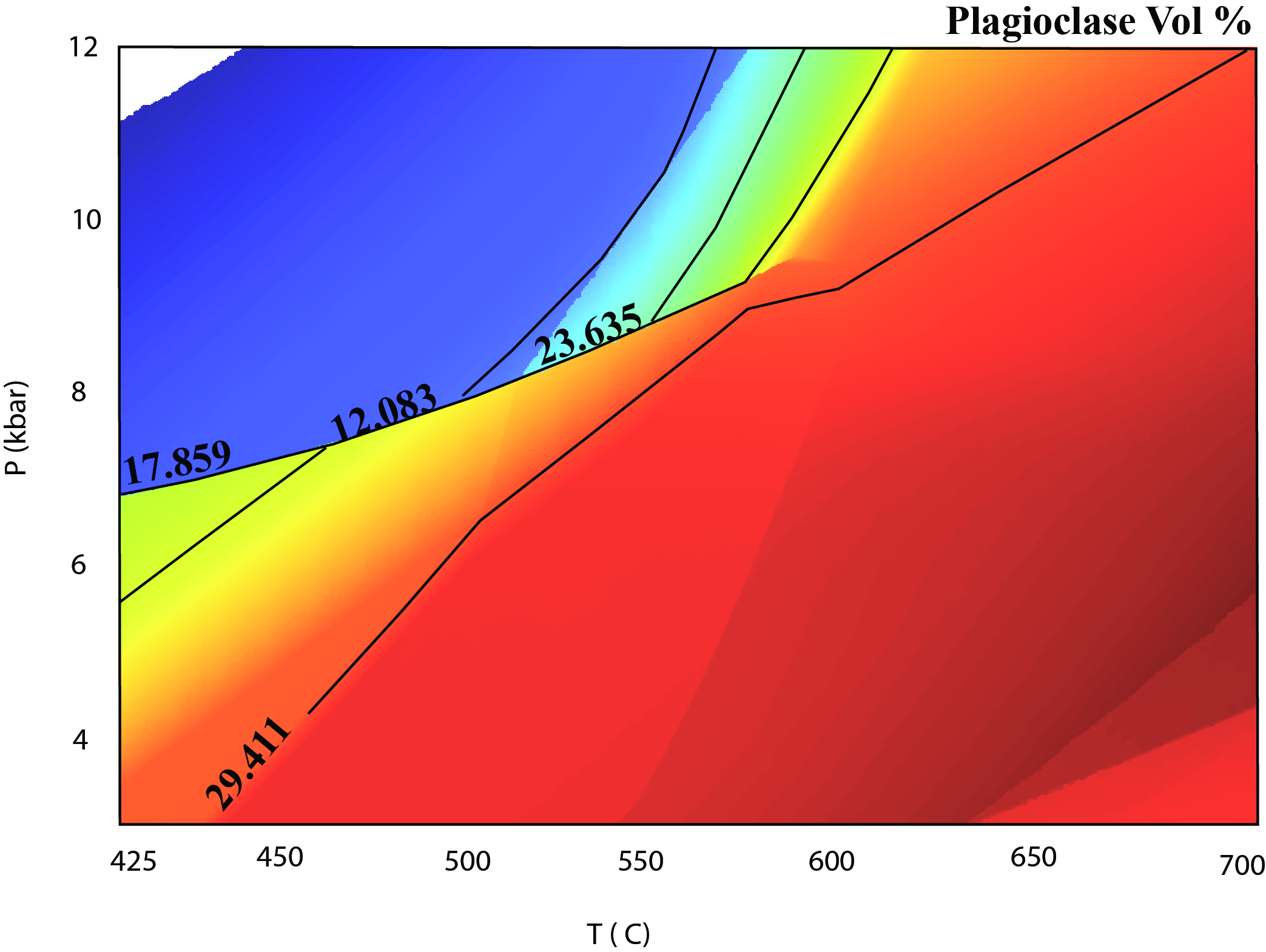

Supplement: Supplementary file 4 [file mmc4.zip › Pseudosections/Pseudosections/D490/isomodes/Plagioclase Vol %.tif]

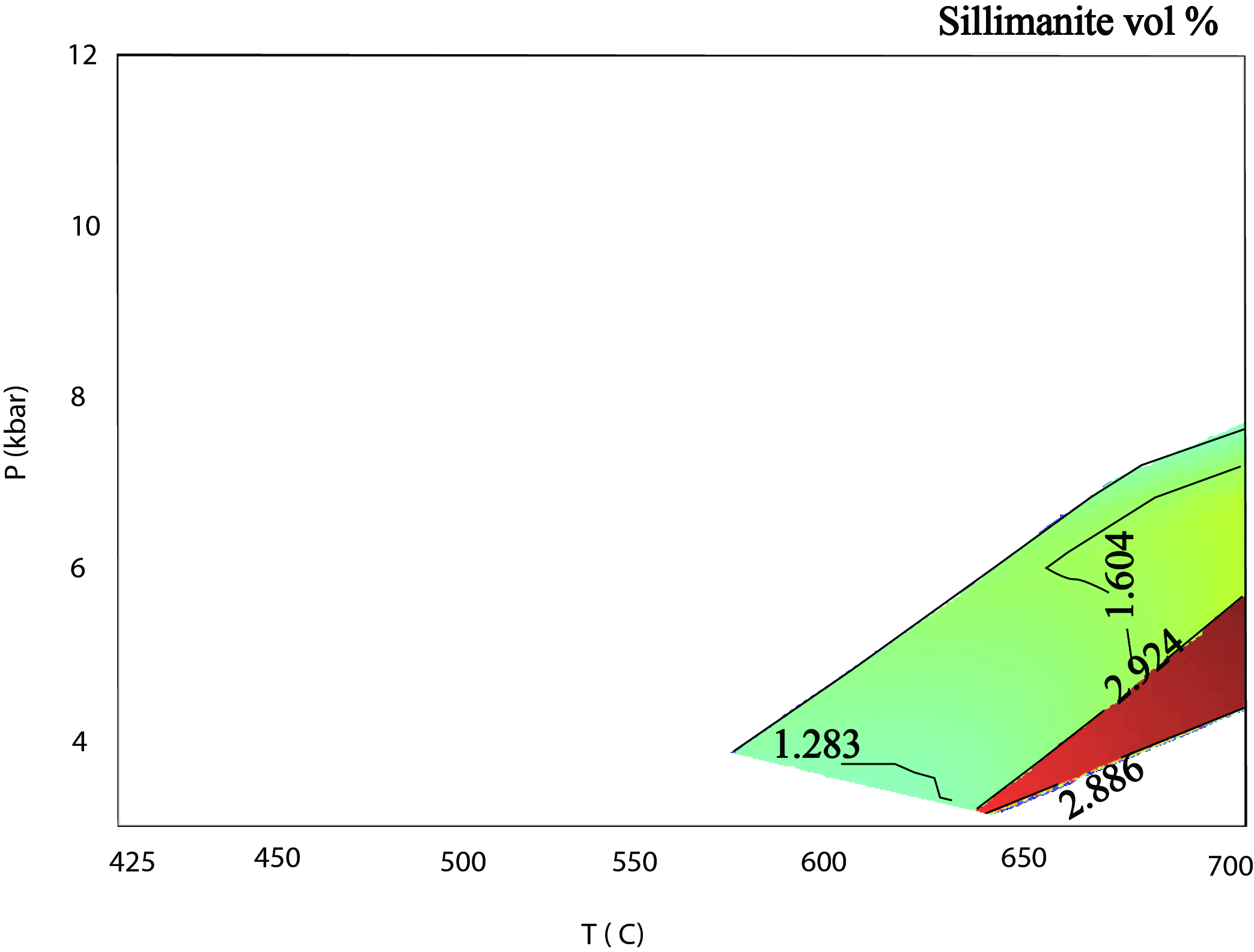

Supplement: Supplementary file 4 [file mmc4.zip › Pseudosections/Pseudosections/D490/isomodes/sillimanite Vol %.tif]

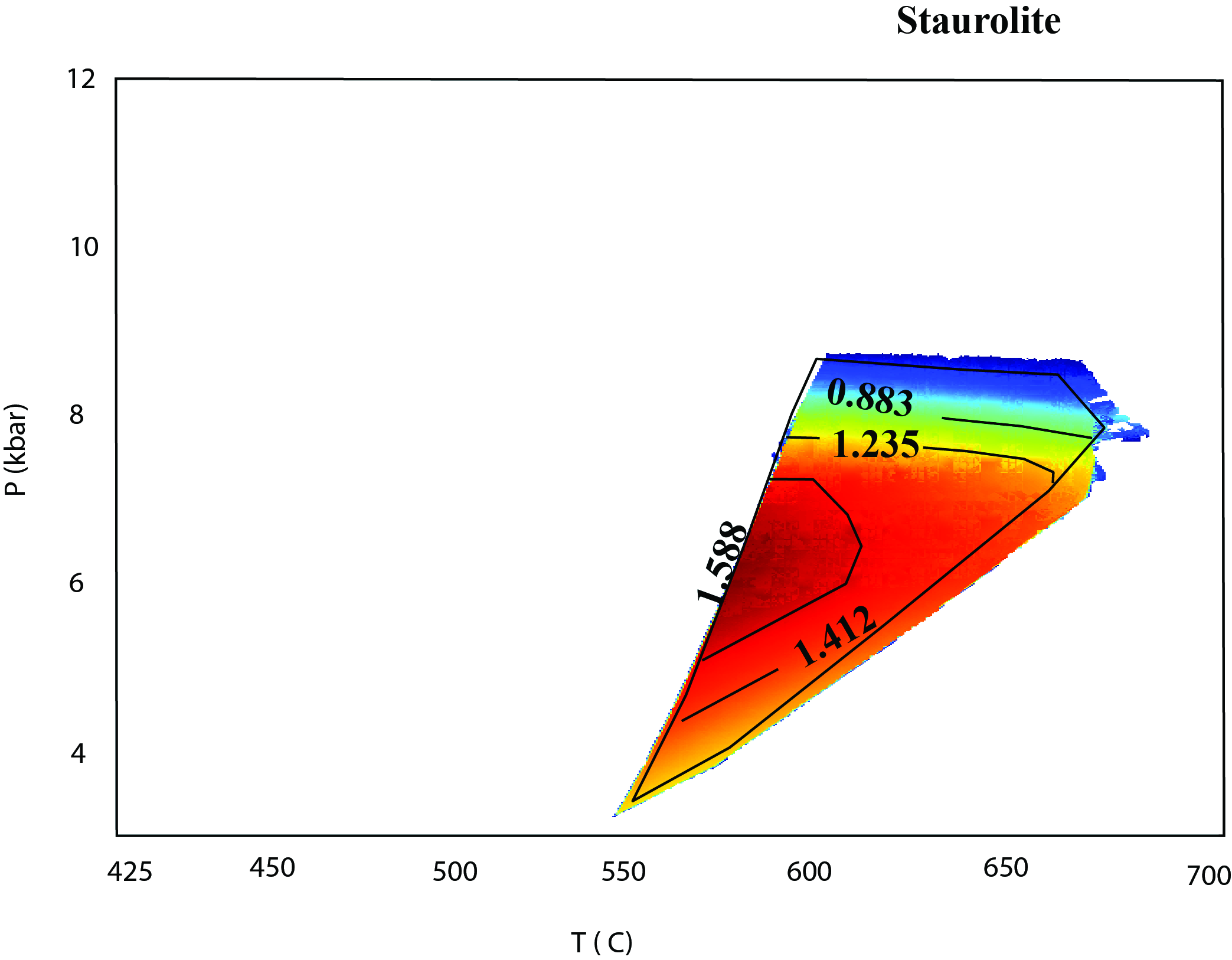

Supplement: Supplementary file 4 [file mmc4.zip › Pseudosections/Pseudosections/D490/isomodes/St edited.tif]

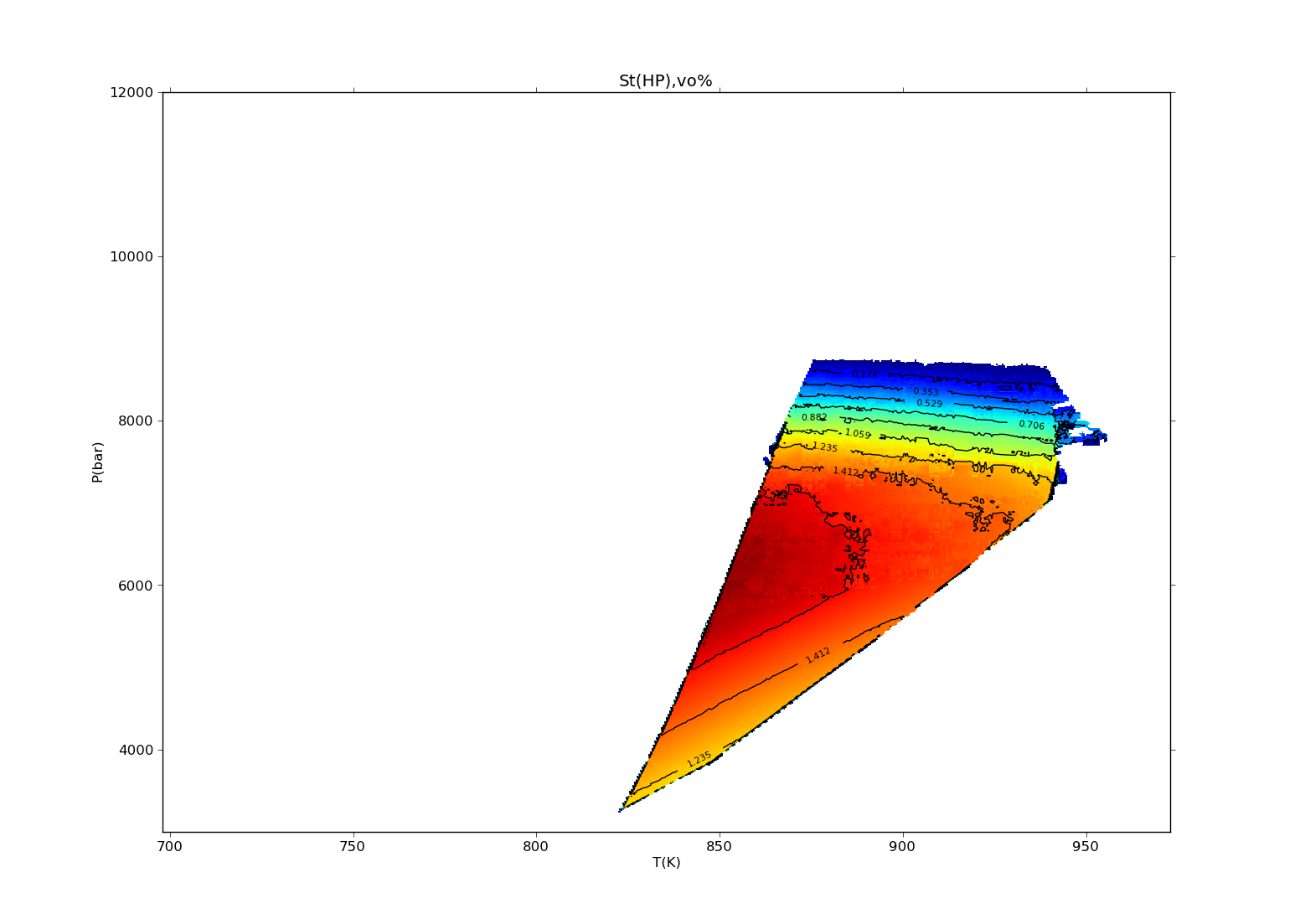

Supplement: Supplementary file 4 [file mmc4.zip › Pseudosections/Pseudosections/D490/isomodes/St.png]

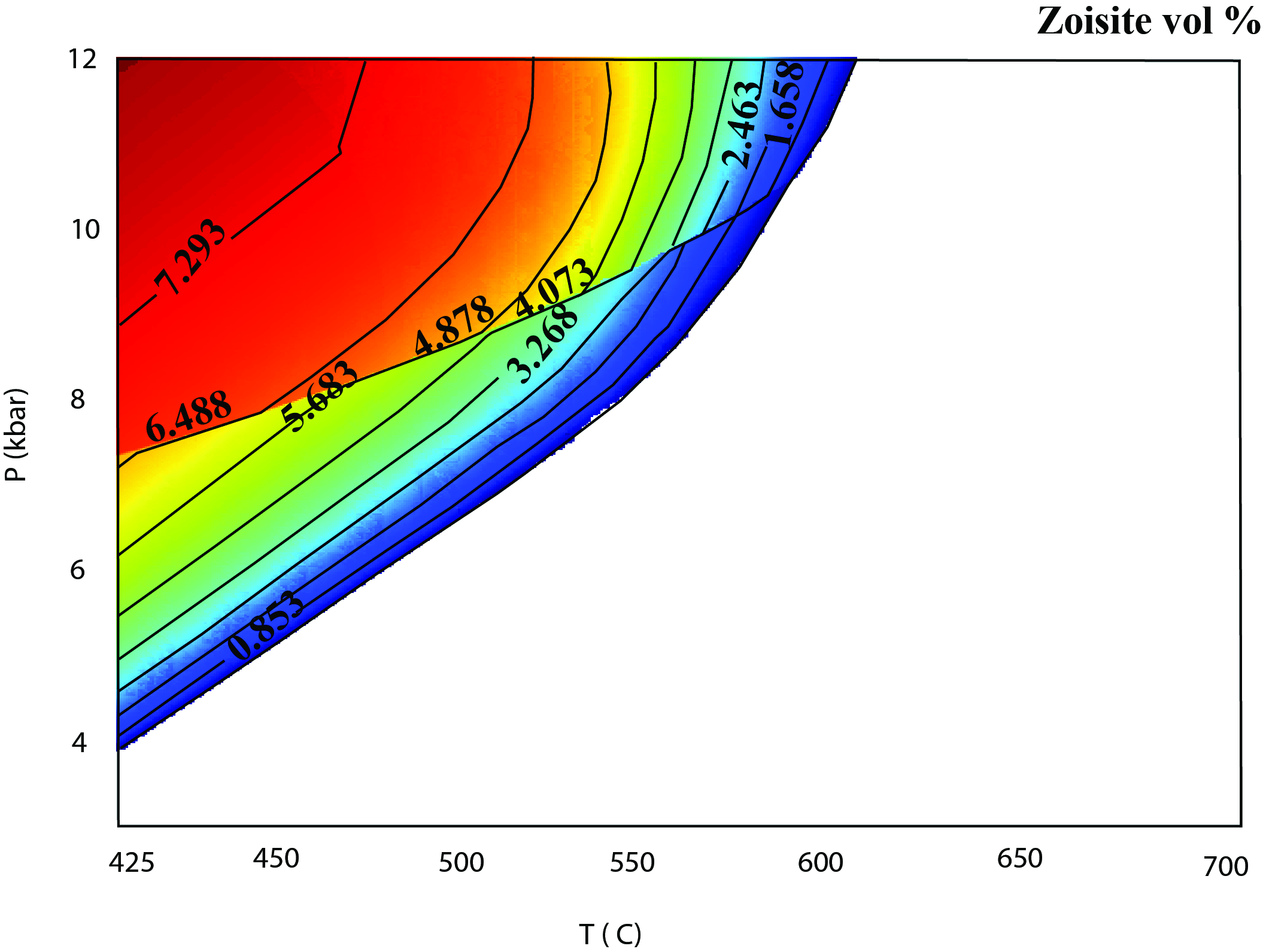

Supplement: Supplementary file 4 [file mmc4.zip › Pseudosections/Pseudosections/D490/isomodes/Zoisite Vol %.tif]

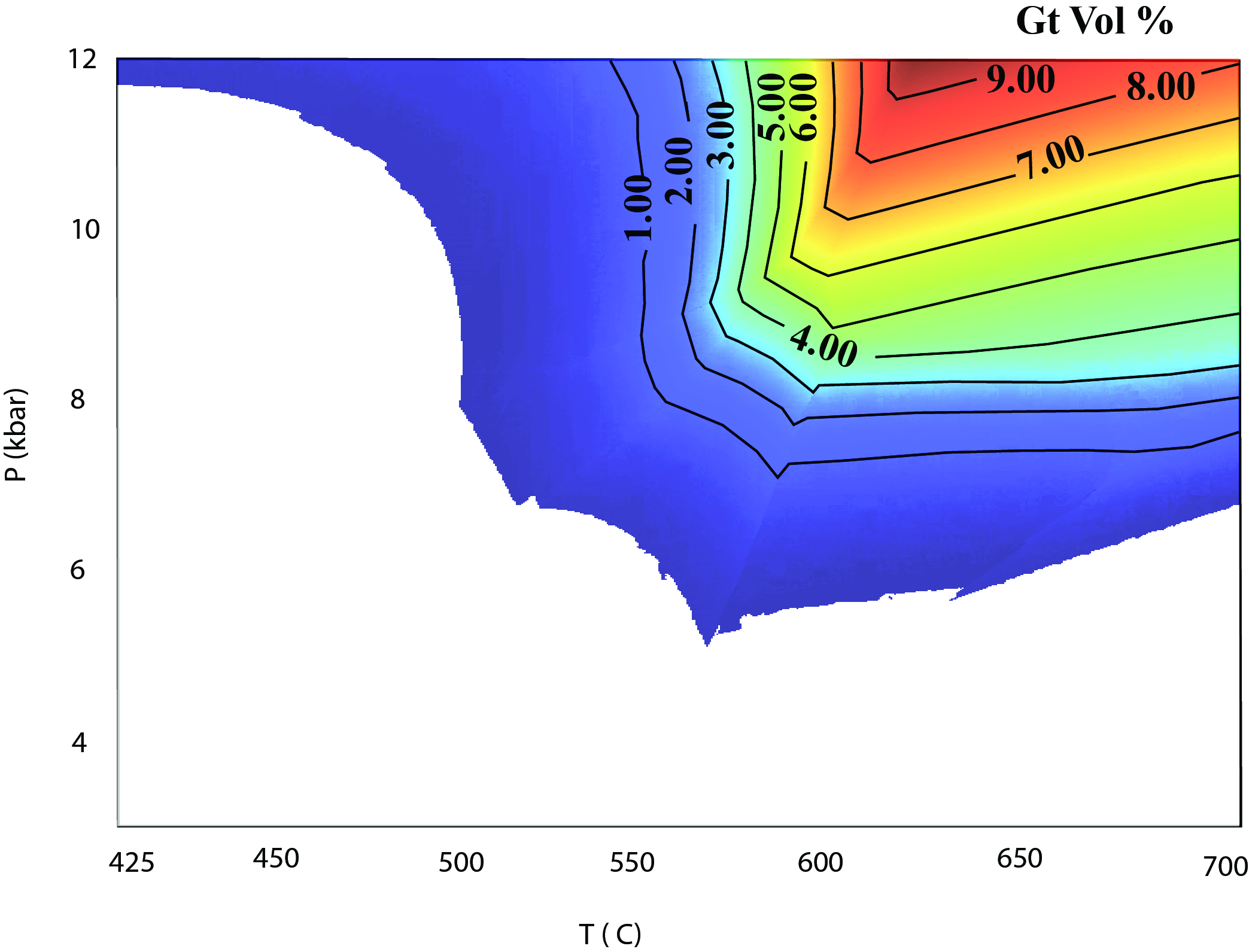

Supplement: Supplementary file 4 [file mmc4.zip › Pseudosections/Pseudosections/D490/isopleths/Garnet Vol %.tif]

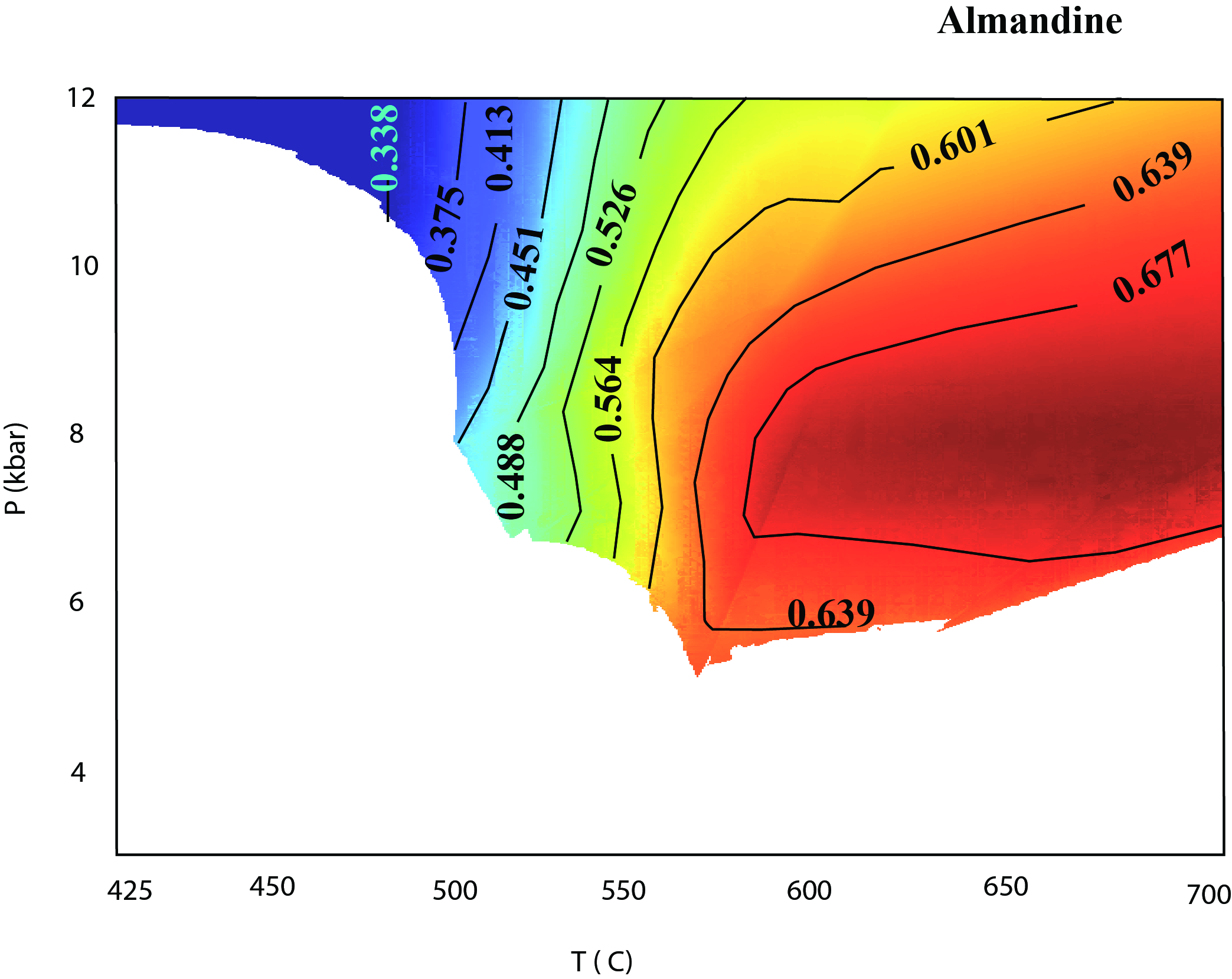

Supplement: Supplementary file 4 [file mmc4.zip › Pseudosections/Pseudosections/D490/isopleths/Garnet_Xalm.tif]

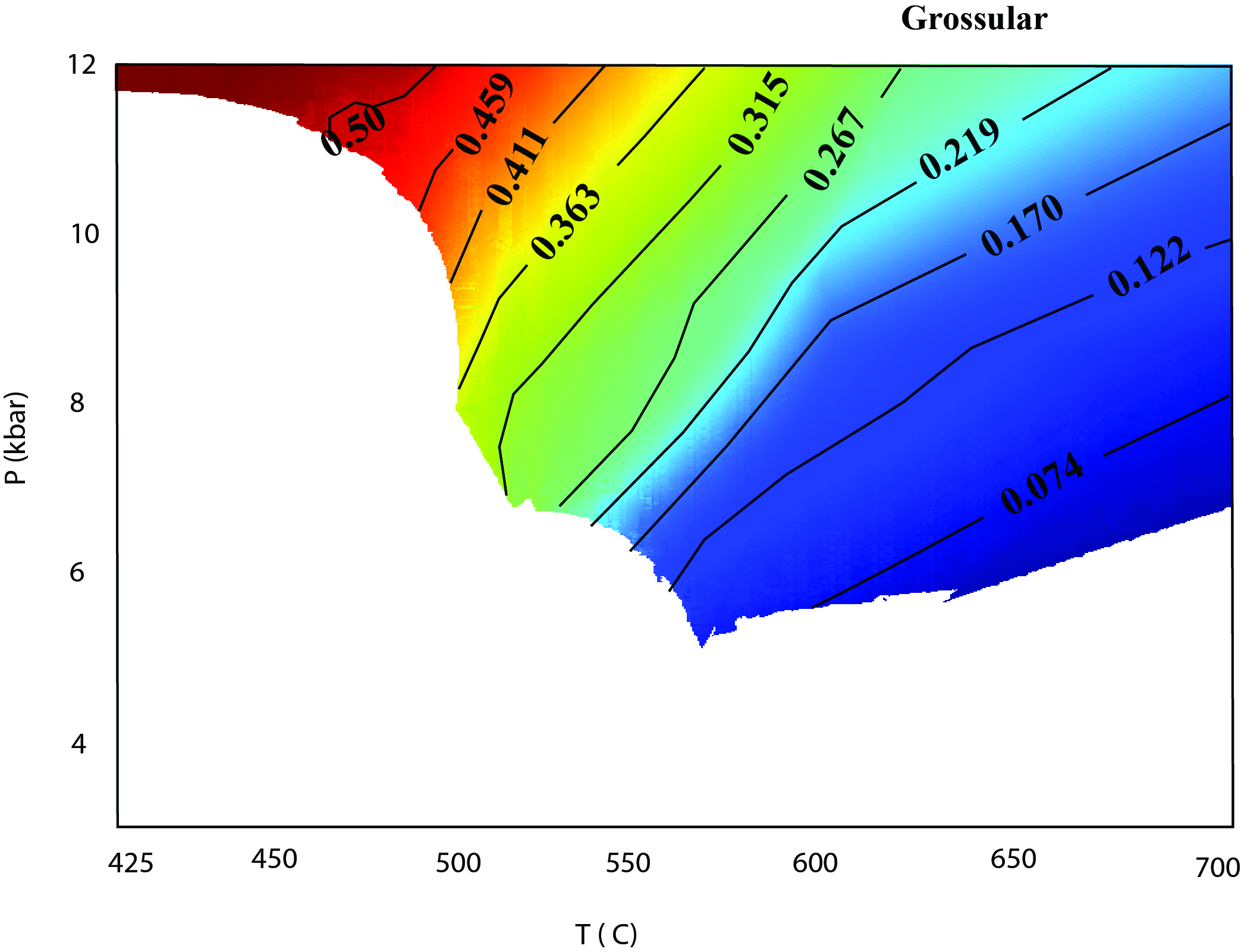

Supplement: Supplementary file 4 [file mmc4.zip › Pseudosections/Pseudosections/D490/isopleths/Garnet_Xgrss.tif]

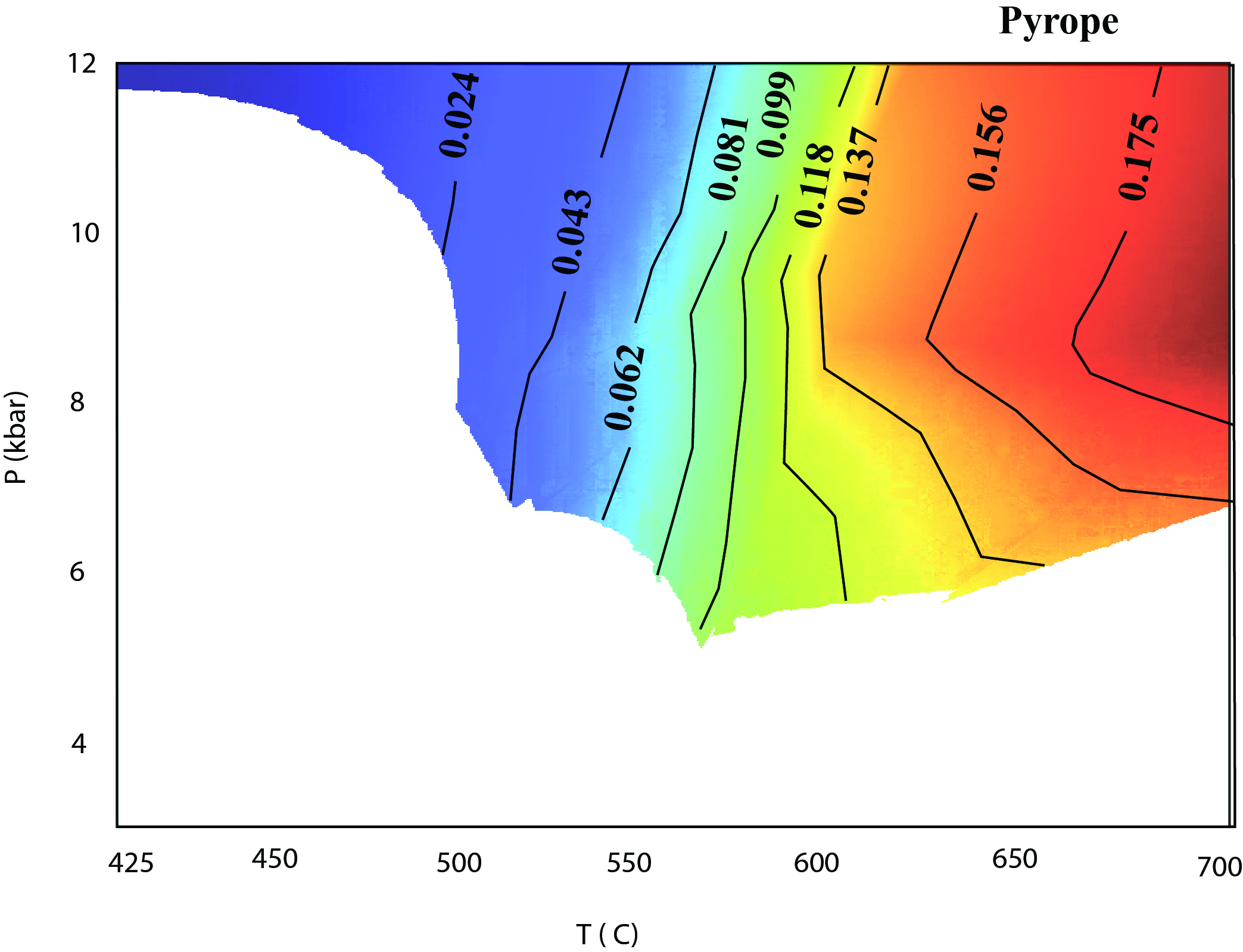

Supplement: Supplementary file 4 [file mmc4.zip › Pseudosections/Pseudosections/D490/isopleths/Garnet_Xpyr.tif]

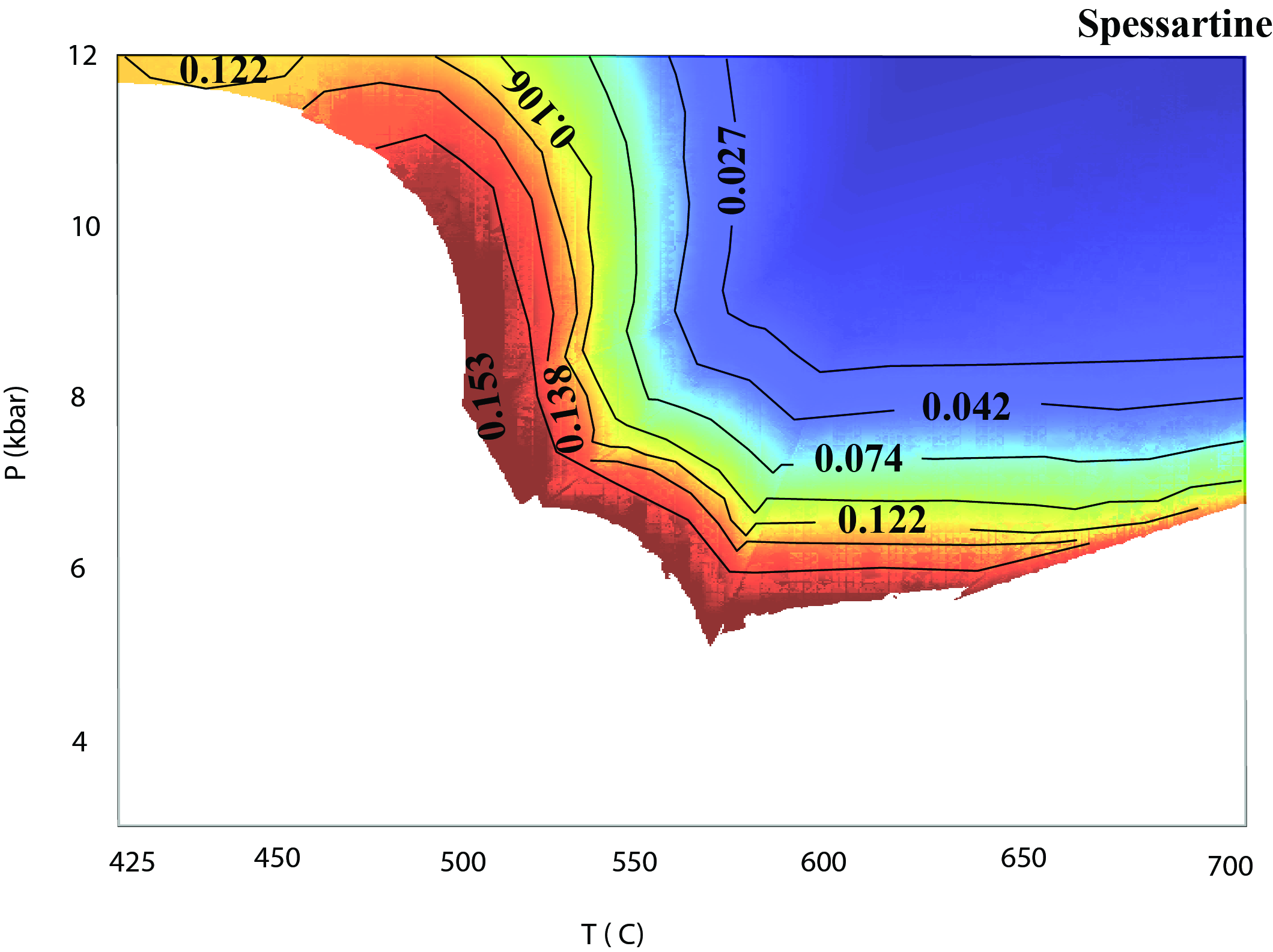

Supplement: Supplementary file 4 [file mmc4.zip › Pseudosections/Pseudosections/D490/isopleths/Garnet_Xsps.tif]

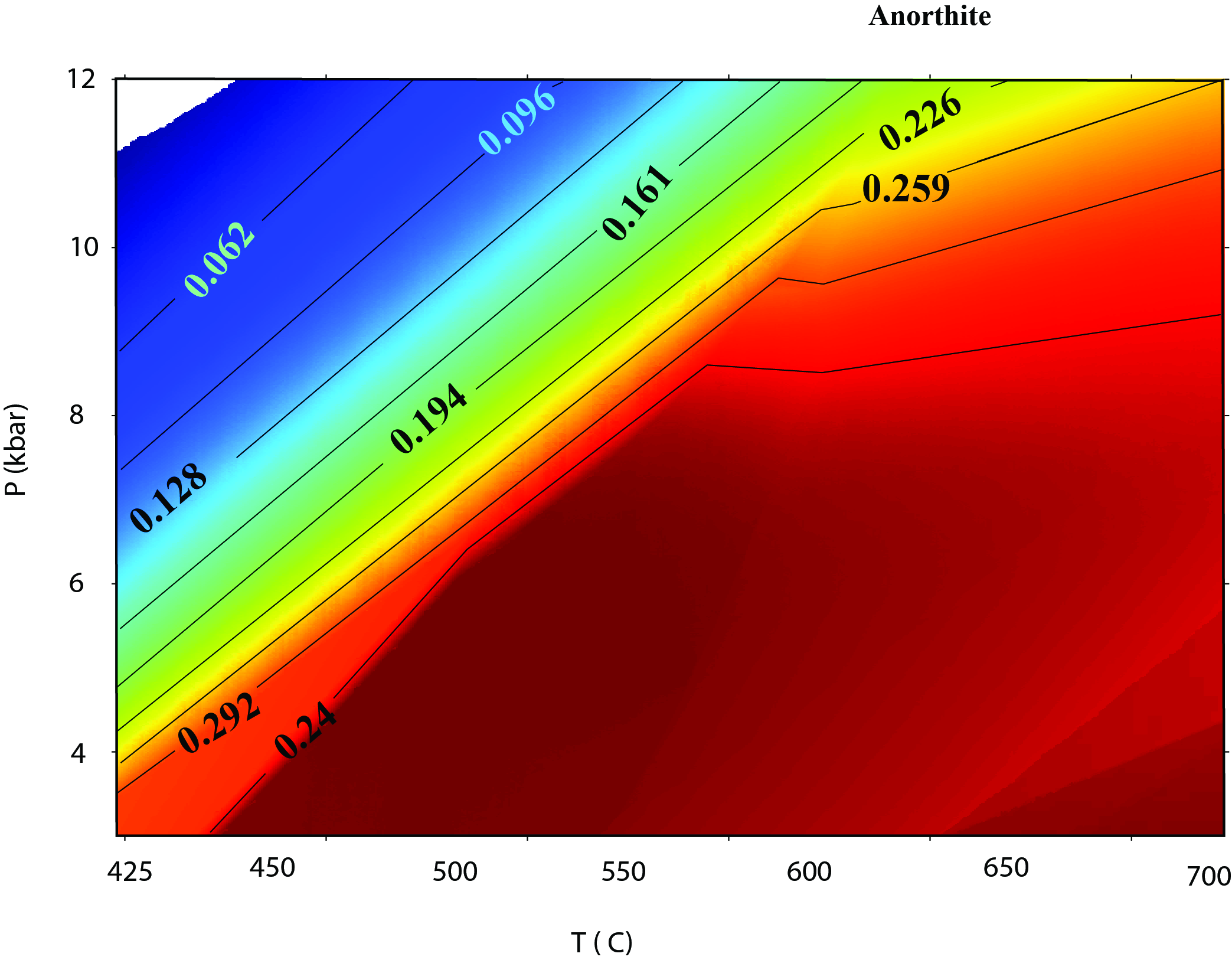

Supplement: Supplementary file 4 [file mmc4.zip › Pseudosections/Pseudosections/D490/isopleths/Plag_XAn.tif]

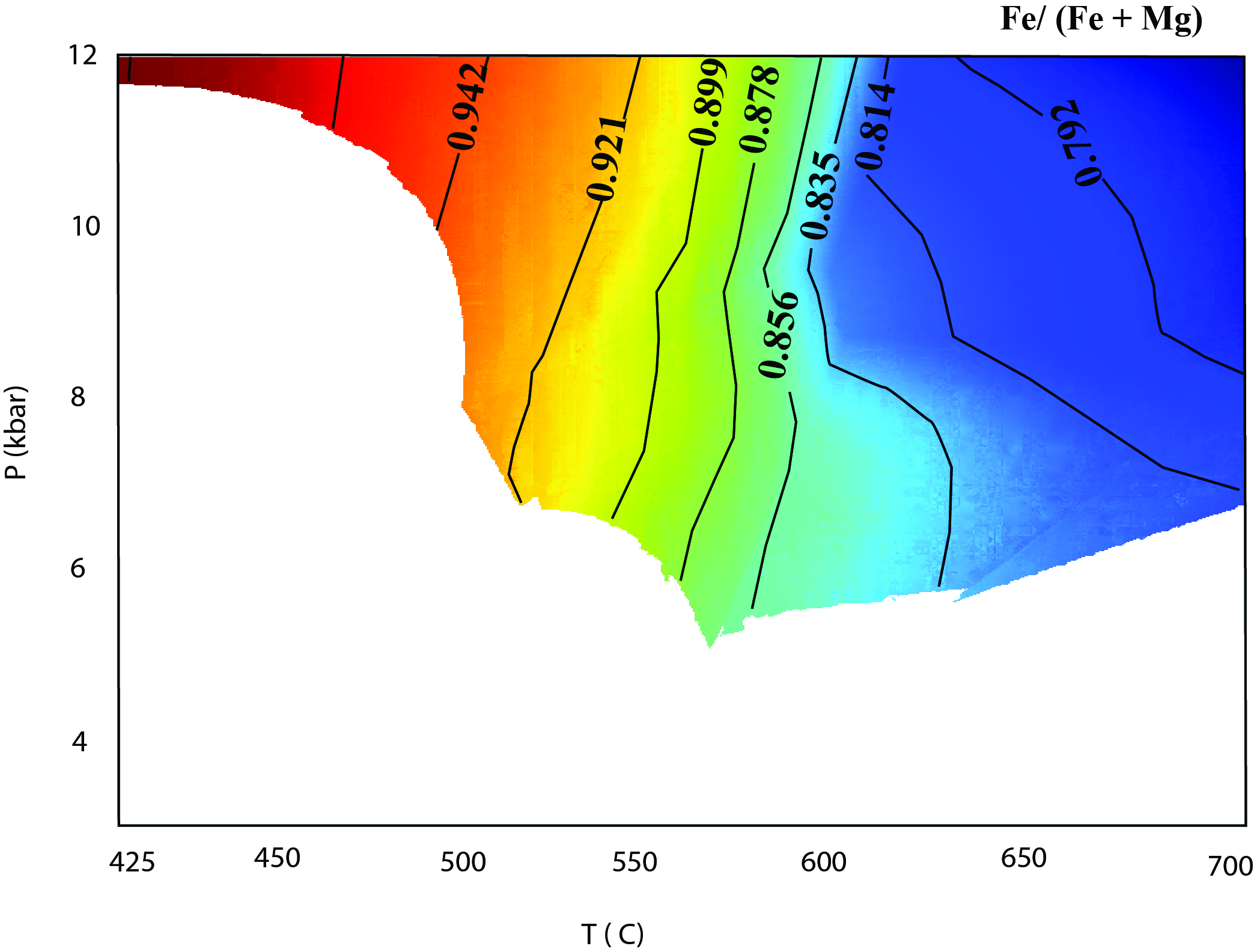

Supplement: Supplementary file 4 [file mmc4.zip › Pseudosections/Pseudosections/D490/isopleths/XFe garnet.tif]
